# Molecular characterization reveals that OsSAPK3 improves drought tolerance and grain yield in rice

**DOI:** 10.1186/s12870-023-04071-8

**Published:** 2023-01-24

**Authors:** Dengji Lou, Suping Lu, Zhen Chen, Yi Lin, Diqiu Yu, Xiaoyan Yang

**Affiliations:** 1 School of Chemical, Biological and Environmental Sciences, Yuxi Normal University, Yuxi, 653100 China; 2 State Key Laboratory for Conservation and Utilization of Bio-Resources in Yunnan, Yunnan University, Kunming, 650091 China; 3 Faculty of Life Science and Technology, Kunming University of Science and Technology, Kunming, 650500 Yunnan China

**Keywords:** Rice, *OsSAPK3*, Drought stress, Grain yield, Tiller, Grain size

## Abstract

**Background:**

Many data suggest that the sucrose non-fermenting 1-related kinases 2 (SnRK2s) are very important to abiotic stress for plants. In rice, these kinases are known as osmotic stress/ABA–activated protein kinases (SAPKs). Osmotic stress/ABA–activated protein kinase 3 (OsSAPK3) is a member of SnRK2II in rice, but its function is still unclear.

**Results:**

The expression of *OsSAPK3* was up regulated by drought, NaCl, PEG and ABA. *OsSAPK3* mutated seedings (*sapk3-1* and *sapk3-2*) showed reduced hypersensitivity to exogenous ABA. In addition, *under drought conditions, sapk3-1* and *sapk3-2* showed more intolerance to drought, including decreased survival rate, increased water loss rate, increased stomatal conductance and significantly decreased expression levels of *SLAC1* and *SLAC7.* Physiological and metabolic analyses showed that OsSAPK3 might play an important role in drought stress signaling pathway by affecting osmotic adjustment and osmolytes, ROS detoxification and expression of ABA dependent and independent dehydration-responsive genes. All gronomic traits analyses demonstrated that *OsSAPK3* could improve rice yield by affecting the regulation of tiller numbers and grain size.

**Conclusion:**

OsSAPK3 plays an important role in both ABA-dependent and ABA-independent drought stress responses. More interestingly, *OsSAPK3* could improve rice yield by indirectly regulating tiller number and grain size. These findings provide new insight for the development of drought-resistant rice.

**Supplementary Information:**

The online version contains supplementary material available at 10.1186/s12870-023-04071-8.

## Background

Rice (*Oryza sativa L.*) is the most important staple crop worldwide. Improving rice yield is an urgent need to solve the crisis of food shortage. Rice yield mainly depends on growth environment, plant height, tillering and other important agronomic traits [1].

Under optimal environmental conditions, rice yield is mainly controlled by tiller numbers per plant, grain yield per plant and thousand-grain weight [1]. Starting with shoot branching, rice tiller experience two stages, axillary meristem formation and tiller bud outgrowth [2]. Therefore, the number and outgrowth rate of tiller buds determine final number of tillers [3]. Previous studies have reported that effective tillers per plant are essential determinant of rice yield, which are affected by genetic and environmental factors [2]. Many genes related to tiller bud formation and outgrowth have been identified in rice, such as *MOC1* [4] and *MOC2* [5]. Earlier studies have also reported that due to the defect of tiller bud formation, *moc1* mutant plants have only a main culm without any tillers [4]. Due to insufficient supply of sucrose, *moc2* mutant plants show significantly reduced tiller numbers, a reduced outgrowth rate and a dwarf phenotype [5]. More interestingly, previous study indicates that tiller enhancer (TE) controls rice tillering by mediating the degradation of MOC1 protein, which encoded an activator of the APC/CTE E3 ubiquitin ligase in rice [6]. In addition, APC/CTE activity is inhibited via phosphorylation of TE by SNF1-related protein kinases (SnRK2s) [7]. These results show that protein kinase plays an important role in regulating rice tillering.

Among environmental stresses, drought is the main threat that affects the development and growth of rice [8]. Drought decreases stomatal conductance, transpiration rate, water use efficiency, relative water content and photosynthesis rate [9]. Under drought conditions, leaf expansion, root growth, plant height and tillering are severely inhibited [10]. All of these morphological and physiological changes are responsible for a reduction in grain yield under drought condition. Drought stress occurring at booting stage [11] (Shao et al., 2014), flowering stage [12] (Liu et al., 2006) and filling stage [13] (Zhang et al., 2018) has larger detrimental influences on rice yield. Water deficit also increases the production of reactive oxygen species (ROS), which leads to peroxidation of lipids, denaturation of proteins, mutation of DNA and various types of cellular oxidative damage [10]. To overcome the damage caused by higher ROS levels, plants produce antioxidants as a tolerance mechanism [10]. Therefore, understanding the mechanism of drought stress response, especially the antioxidant mechanism under drought stress, will help to improve crop productivity under drought stress.

Numerous reports show that protein kinases involve in plant responses to biotic and abiotic stresses. With the ability to phosphorylate specific substrates, protein kinases are key components in plant drought stress response [14]. As plant specific protein kinases, SnRK2 kinases (SnRK2s) have been found in many seed plants, such as rice, maize, tobacco, tomato, wheat and soybean [15]. According to the conservation of sequences and active domains, SnRK2s are divided into three subclasses [11]. In *Arabidopsis thaliana*, SnRK2.1, 2.4, 2.5, 2.9, and 2.10 belong to groupI, SnRK2.7 and 2.8 to group II, and SnRK2.2, 2.3, and 2.6 to group III. In rice, these kinases are designated as osmotic stress/ABA–activated protein kinases (SAPKs). SnRK2I includes SAPK4-SAPK7, SnRK2II includes SAPK1-SAPK3, and SnRK2III includes SAPK8-SAPK10 [16]. Among SnRK2s, subclass III members act as key positive regulators in ABA signaling, which regulate the expression of stress-responsive genes in an ABA-dependent manner [17, 18]. In *A. thaliana*, three subclass III SnRK2s (SnRK2.2, 2.3, and 2.6) are mainly involved in ABA-dependent drought stress signaling. The *snrk2.2/snrk2.6/snrk2.3* triple mutant shows severe ABA-insensitive viviparity and drought-sensitive phenotype [19–22]. In rice, members of SnRK2 subclass III (SAPK8–10) are involved in osmotic stress response by regulating ABA-dependent gene expression [23–25]. The protein phosphorylation network of subclass III SnRK2 has also been elucidated, which involves mitogen-activated protein (MAP) kinase (MAPK), epigenetic regulation and RNA processing [18, 26]. Subclass I SnRK2s have been identified as playing important roles in early processes of drought stress [27–30]. For example, *SnRK2.4* and *SnRK2.10* are activated by ABI1 and PP2CA under osmotic stress in *A. thaliana* [27, 28]. OsSAPK6 can phosphorylate and activate OsbZIP10 and OsbZIP46 in the ABA signaling pathway [29, 30]. For subclass II SnRK2s, SnRK2.8 positively regulates drought resistance response and participates in root growth [31]. A recent report showed that SnRK2.7 and SnRK2.8 regulated drought response genes and transcription factors bound to ABA response elements in *A. thaliana* [32]. Recent report indicated that SAPK2 is involved in the osmotic stress response by phosphorylating OsbZIP23 and OsbZIP46 and promoting the transcription of stress response genes [33, 34]. These data indicate that SnRK2s are crucial for abiotic stress responses in rice. Therefore, it is necessary to clarify the special function of SnRK2s.

Up till now, the function of *OsSAPK3* has not been reported yet. In this study, *sapk3* mutants (*sapk3-1* and *sapk3-2*) generated by CRISPR/Cas9 system were used to characterize the role of *OsSAPK3* in responding to drought stress and regulating rice yield. These results provide new insight for functional analysis of SnRK2s and engineering of drought resistant rice.

## Results

### Cloning and sequence analysis of *OsSAPK3*

**Fig. 1:**
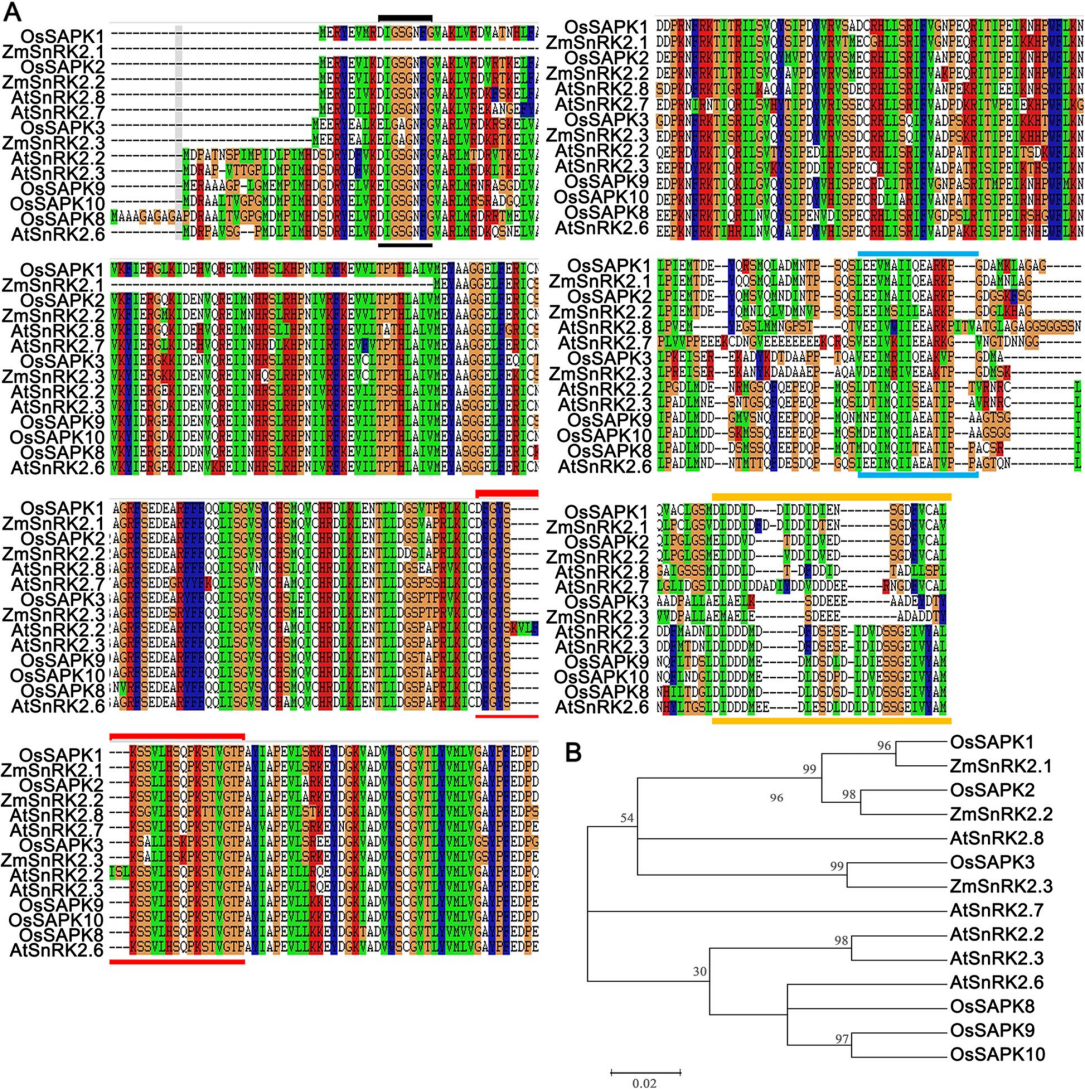
Sequence analysis and phylogenetic relationship of SnRK2II and SnRK2III subfamily genes. **A** Amino acids comparison and **B** Phylogenetic relationship of selected SnRK2 members in *Arabidopsis thaliana* (At), *Oryza sativa* (Os) and *Zea mays* (Zm). The ATP-binding domain (ATP) and the activation loop are marked with black and red lines respectively. The SnRK2s conserved motif and the ABA-activated SnRK2s motif (ABA box) are marked with blue and yellow lines respectively

The open reading frame of Os*SAPK3* consists of 1,005 bp, which encoded 334 amino acids (Annotation identified in Introduction to the Rice Genome Annotation Project. database:LOC_Os10g41490 http://rice.plantbiology.msu.edu/index.shtml). SnRK2s contain a Ser/Thr enzyme activity conservative domain DFGYSKSSVLHSQPKSTVGTPAYIAPE and an ATP binding site GXGXXGX. The C-terminal sequence has high polymorphism, mainly including response to osmotic and ABA stress [16]. *OsSAPK1*, *OsSAPK2* and *OsSAPK3* are all rice SnRK2 II subfamily members. Similar to SnRK2 III members that play an important role in ABA pathway, *OsSAPK3* contained several conservative domains, such as an ATP binding domain (ATP) (marked with black), the activation loop (marked with red lines) and SnRK2s conserved motif (marked with blue lines) (Fig. 1 A, Supplementary Table 1). But there is a significant difference in the ABA-activated motif (ABA box) (marked with yellow lines) comparing with ABA-pathway SnRK2s (Fig. 1 A, Supplementary Table 1).

To analyze the closeness of Os*SAPK3* to ABA dependent SnRK2 III members, a phylogenetic tree was constructed based on their amino acids sequence of selected SnRK2 II and SnRK2 III members from *A. thaliana* (At), *Oryza sativa* (Os) and *Zea mays* (Zm) (Supplementary Table 1). These results showed that *OsSAPK3* was similar to *ZmSAPK3* (Fig. 1 B). In addition, it is highly similar to *AtSnRK2.8* in *A. thaliana* and *OsSAPK2* in rice (Fig. 1 B). These results indicated that *OsSAPK3* might have functions different from subclass III SnRK2s but similar to *OsSAPK2* and *AtSnRK2.8*.

### Expression profile and localization of *OsSAPK3*

**Fig. 2:**
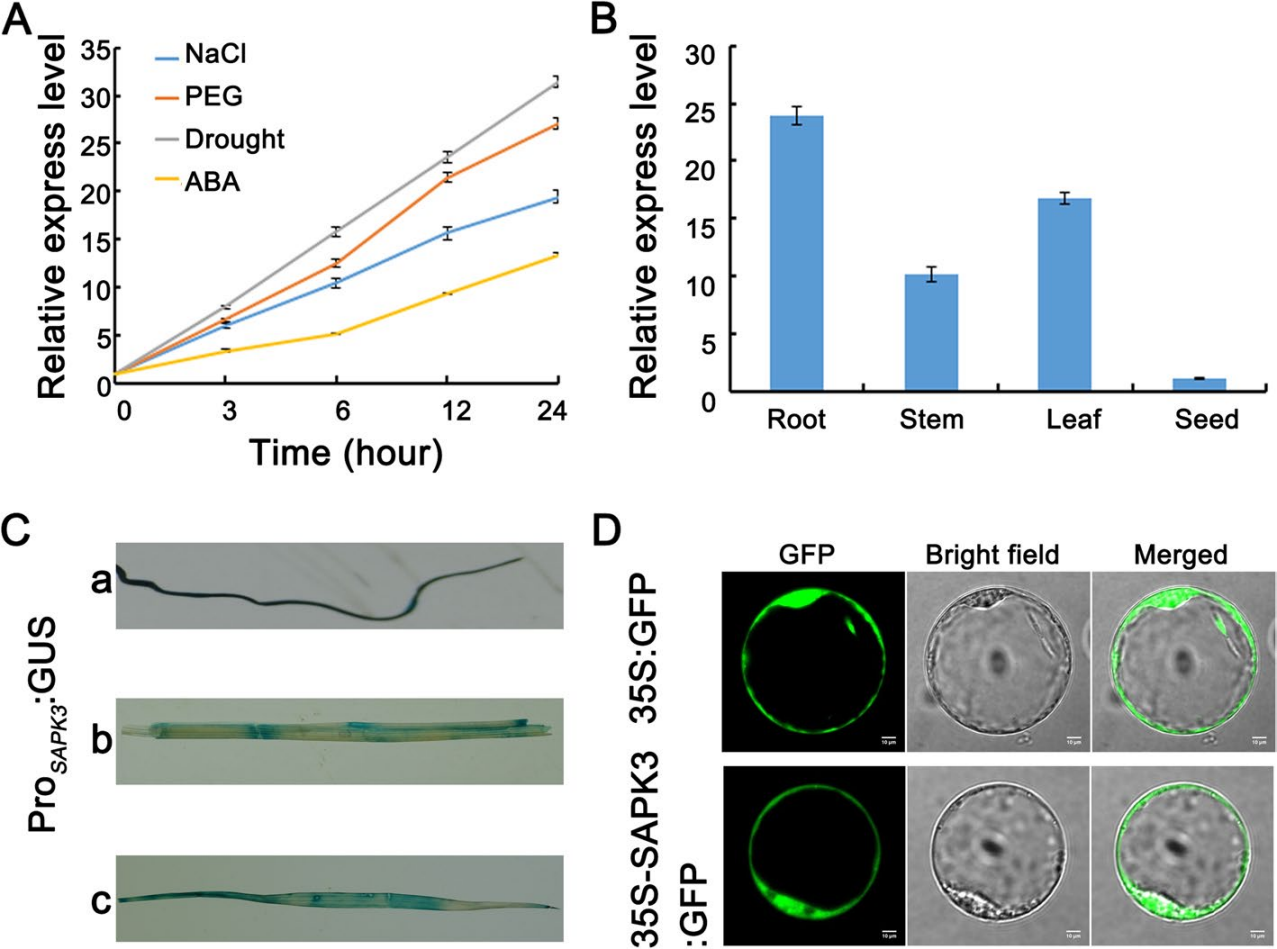
The expression pattern of *OsSAPK3* **A** Transcription analyses of *OsSAPK3* under various stress, such as 150 mM NaCl, 20% PEG6000, exposed to the air or 100 μM ABA. **B** Transcription analyses of *OsSAPK3* in different rice tissues by qRT-PCR. **C** GUS staining analyses of Pro_SAPK3_-GUS expression in different rice tissues. a, root; b, stem; c, leaf. (D) Subcellular localization of *OsSAPK3* in rice protoplasts. Scale bar = 10 μm. Data represent means ± SD of three biological replicates (5 plants for each replicate)

To analyze the responsiveness of *OsSAPK3* under different abiotic stresses in rice, *OsSAPK3* expression profiles were conducted via quantitative RT-PCR (qRT-PCR). Under drought, NaCl and PEG treatments, the expression of *OsSAPK3* remarkably increased (Fig. 2 A). In ABA-treated plants, *OsSAPK3* expression was weakly induced (Fig. 2 A).These results indicated that *OsSAPK3* played a role in osmotic stress response, but had moderate role in ABA tolerance.

To investigate the biological role of Os*SAPK3*, the spatial and temporal expression of Os*SAPK3* in different tissues were analyzed. The qRT-PCR results displayed that Os*SAPK3* expression level was highest in roots, followed by leaves (Fig. 2 B). To further verify this result, Pro_*OsSAPK3*_-Gus plants were used for detecting tissue expression patterns. The GUS activity was relatively higher in roots than in leaves (Fig. 2 C), which was consistent with qRT-PCR analyses. The subcellular localization of *OsSAPK3* was assessed through transient expression of the *OsSAPK3*-GFP fusion protein in rice protoplasts. Fluorescence microscopy analyses showed that *OsSAPK3* was localized in the nucleus and cytoplasm (Fig. 2 D).

### *OsSAPK3* mutated plants show decreased ABA sensitivity

To explore the function of *OsSAPK3* in responses to ABA stress, *OsSAPK3* mutated plants were generated using CRISPR/Cas9 gene editing technology. Two homozygous lines were chosen for ABA tolerance evaluation, which were named *sapk3-1* and *sapk3-2*.

**Fig. 3:**
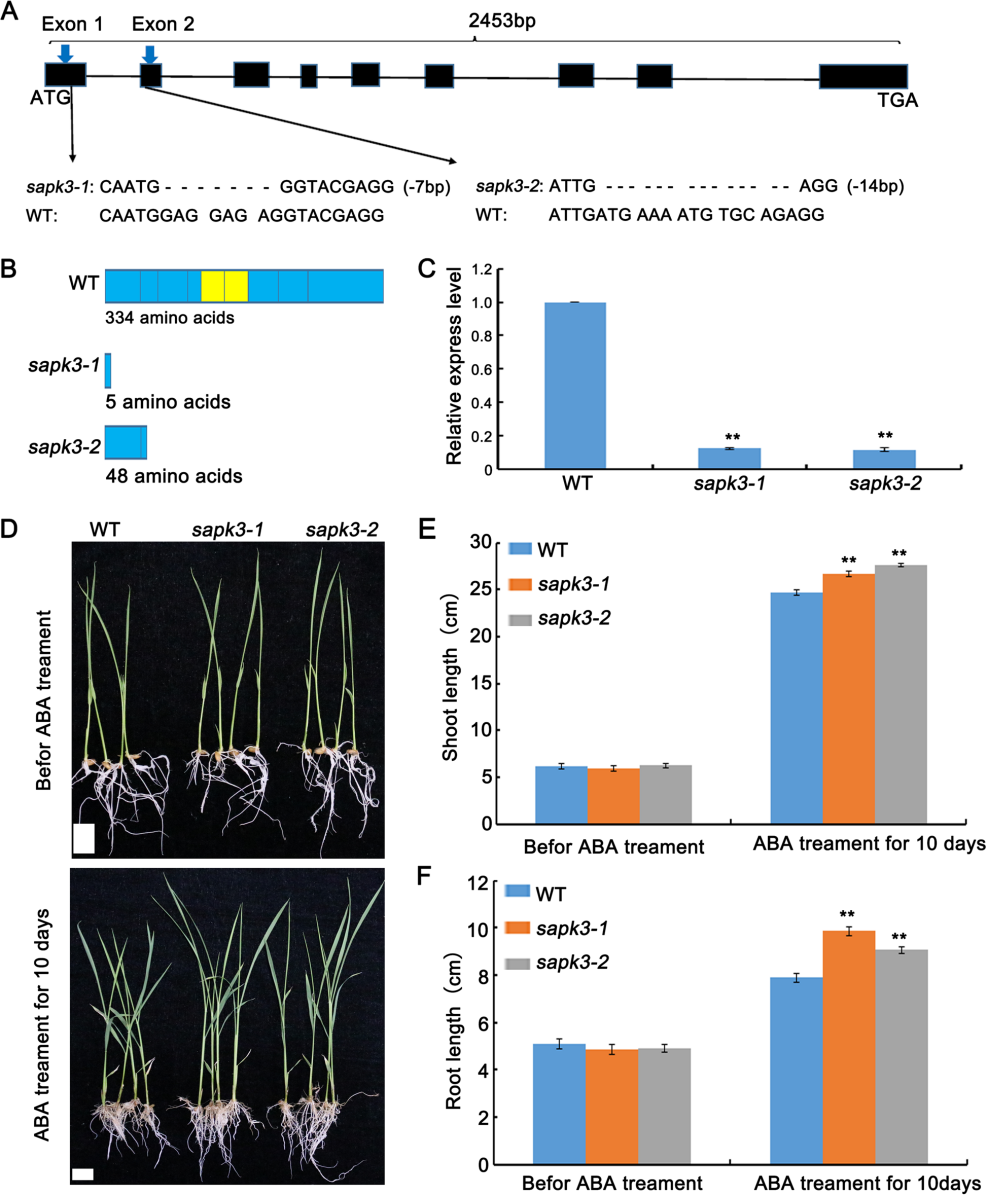
*OsSAPK3* mutated plants show decreased ABA sensitivity. **A** Sequence comparison of wild-type plants and two *sapk3* mutant lines. **B** Protein structures of wild-type plants and two *sapk3* mutant lines. **C** Transcript accumulation of *OsSAPK3* in wild-type plants and two *sapk3* mutant lines. **D** Phenotypes of wild-type plants and two *sapk3* mutant lines before and after 100 μM ABA treatment for10 days. Scale bars = 2 cm. **E** Shoot and **F** Root length corresponding to D. Data represent means ± SD of three biological replicates (16 plants for each replicate). **P < 0.01 (Student’s t-test)

The *sapk3-1* plants contained a 7-bp deletion in the first exon of *OsSAPK3*, while *sapk3-2* plants carried a 14-bp deletion in second exon (Fig. 3 A, Supplementary Table 2). Amino acid sequence analyses showed that OsSAPK3 protein from *sapk3-1* and *sapk3-2* mutant plants had only 5 or 48 amino acids due to premature termination of translation. (Fig. 3 B, Supplementary Table 2). In addition, *OsSAPK3* expression level was much lower in *sapk3-1* and *sapk3-2* plants than in wild-type plants (Fig. 3 C). These results indicated that *sapk3-1* and *sapk3-2* mutant lines were *OsSAPK3* loss-of-function mutants.

In order to determine whether OsSAPK3 participates in ABA responses, shoot and root length of the wild-typeplants and two *sapk3* mutant lines were examined after treatment with 100 μM exogenous ABA for 10 days. Compared with wild-type plants, the shoot and root growth inhibition significantly reduced in two *sapk3* mutant lines after ABA treatment (Fig. 3 D-F), which indicated that *sapk3-1* and *sapk3-2* plants were not sensitive to ABA. These results demonstrated that *OsSAPK3* played a role in ABA dependent signaling pathway.

### *OsSAPK3* mutated plants show reduced drought stress tolerance

**Fig. 4:**
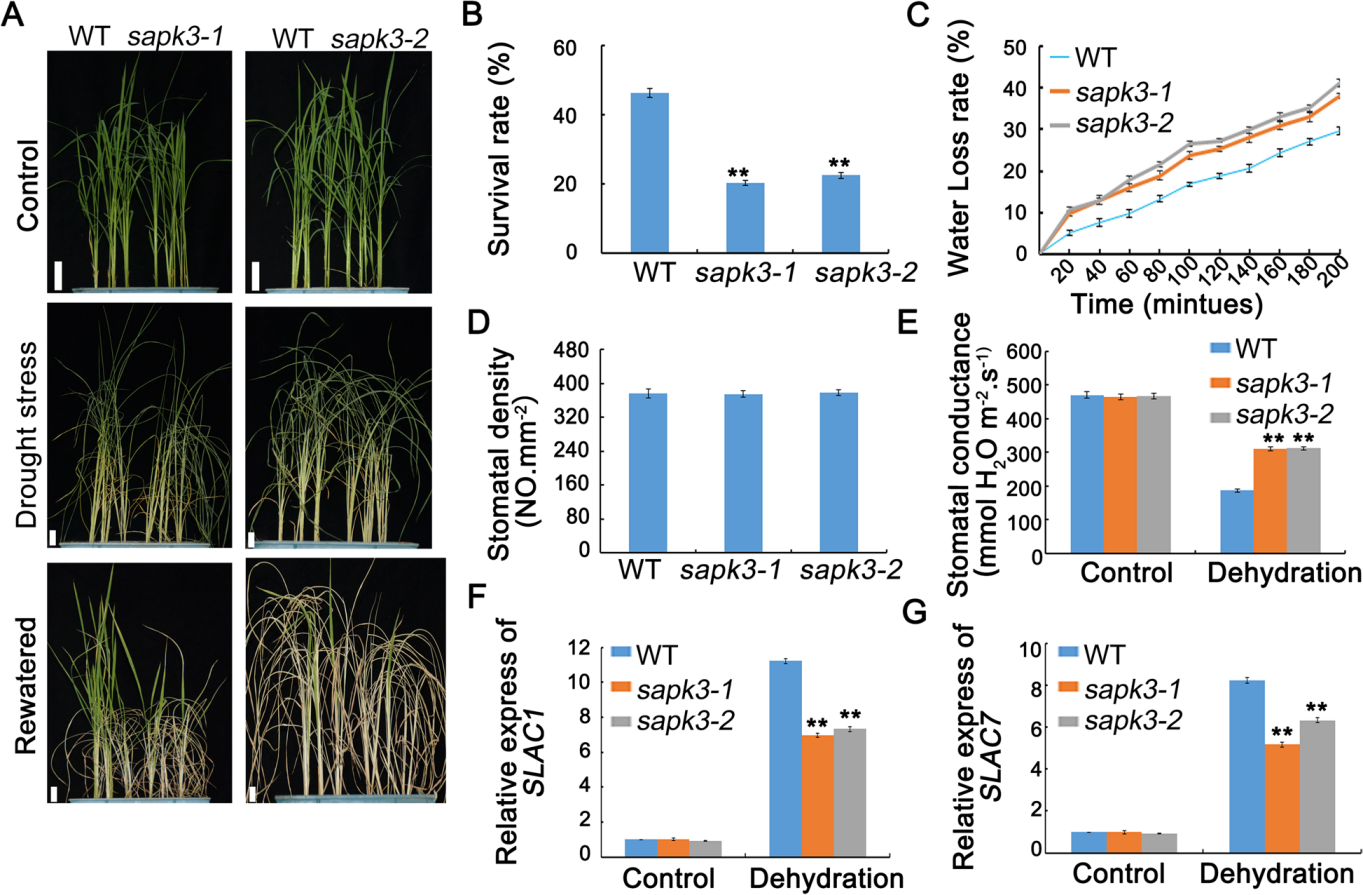
*OsSAPK3* mutated plants show reduced drought stress tolerance **A** Phenotype of wild-type plants and two *sapk3* mutant lines before and after drought stress treament for 7 days. Scale bars = 2 cm. **B** Survival rates corresponding to A. **C** Relative water loss rate of leaves from 2-week-old wild-type plants and two *sapk3* mutant lines under drought stress. **D** Stomatal density and **E** Stomatal conductance from wild-type plants and two *sapk3* mutant lines before and after drought stress. **F** Transcript level of stomata genes *OsSLAC1* and **G** *OsSLAC7* in wild-type plants and two *sapk3* mutant lines before and after drought stress. Values represent the means ± SD of three biological replicates (5 plants for each replicate). **P < 0.01 (Student’s t-test)

To confirm the function of *OsSAPK3* in drought tolerance in rice, the performance of wild-type plants and two *sapk3* mutant lines under drought stress was examined by withholding water for 7 days. The survival rate of wild-type plants (46.4%) was significantly higher than that in two *sapk3* mutant lines (i.e., *sapk3-1* and *sapk3-2* plants survival rates were only 20.4% and 22.5%, respectively) (Fig. 4 A and B). This result demonstrated that *OsSAPK3* played a positive role in drought stress response.

Since the drought tolerance is closely related to relative water loss rate, the relative water loss rate was observed. As shown in Fig. 4, the relative water loss rate of wild-type leaves was higher than that in two *sapk3* mutant lines (Fig. 4 C). Stomatal status is important for drought tolerance, then the stomatal density and stomatal conductance at 6-leaves stage were examined. There was no obvious difference for stomatal density between the wild-type plants and two *sapk3* mutant lines (Fig. 4 D). Under normal conditions, the stomatal conductance of all plants had no significant difference between wild-type plants and *sapk3* mutants. However, stomatal conductance of wild-type plants was significantly lower than that in two *sapk3* mutant lines under drought conditions (Fig. 4 E). Based on these results, *OsSAPK3* may change the stomatal conductance by participating in ABA dependent stomatal regulation. To test this assumption, the expression levels of *slow anion channel-associated 1* (*OsSLAC1*) and *slow anion channel-associated 7* (*OsSLAC7*) were verified. The expression levels of these two genes in two *sapk3* mutant lines were significantly lower than that in wild-type plants under drought conditions (Fig. 4 F and D).

These results indicated that *OsSAPK3* improved the tolerance to drought stress by regulating stomatal conductance through regulating the expression of *OsSLAC1* and *OsSLAC7*.

### Metabolic regulation of *sapk3* mutants in response to drought stress

**Fig. 5:**
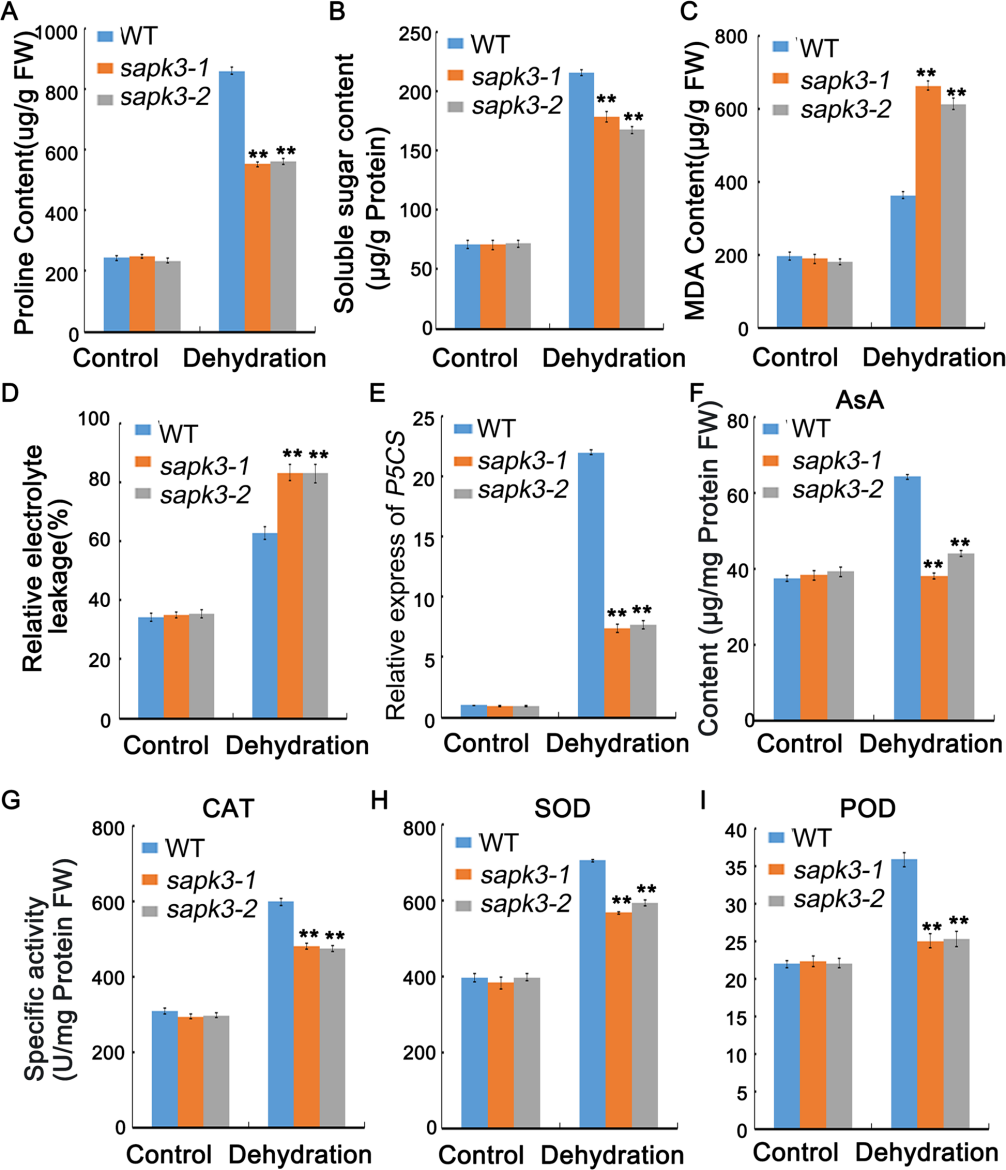
Metabolic regulation of *sapk3* mutants in response to drought stress. **A** Proline **B** Soluble sugar **C** MDA contents in the seedlings of wild-type plants and two *sapk3* mutant lines before and after drought stress treament for 7 days. **D** Relative ion leakage in wild-type plants and two *sapk3* mutant lines leaves before and after drought stress treament for 7 days. **E** Relative expression levels of *OsP5CS* in the seedlings of wild-type plants and two *sapk3* mutant lines before and after drought stress treament for 7 days. **F** AsA content in the seedlings of wild-type plants and two *sapk3* mutant lines before and after drought stress treament for 7 days. **G** CAT **H** SOD **I** POD activities in the seedlings of wild-type plants and two *sapk3* mutant lines before and after drought stress treament for 7 days. Values represent the means ± SD of three biological replicates (5 plants for each replicate). **P < 0.01 (Student’s t-test). FW: Fresh weight

Plants adapt to drought stress through physiological and biochemical changes, such as the accumulation of proline and soluble sugar [23]. Thus, the content of proline and soluble sugar was examined. Under normal conditions, there is no significant difference in proline and soluble sugar content among all plants (Fig. 5 A and B). After drought treatment, the proline and soluble sugar content in two *sapk3* mutant lines were significantly lower than that in wild-type plants (Fig. 5 A and B). Proline biosynthesis is catalyzed by 1-pyrroline-5- carboxylate synthetase (*OsP5CS*) [35]. According to qRT-PCR data, the *OsP5CS* expression level was much lower in *sapk3* mutants than in wild-type plants under drought conditions (Fig. 5 E), which was consistent with proline content. These results suggested that *OsSAPK3* might positively regulate the resistance to drought stress via changing the accumulation of osmolyte, such as proline and soluble sugars.

### *OsSAPK3* affects ROS detoxification via regulating expression of antioxidant genes under drought conditions

Plants under abiotic stress generate various kinds of ROS molecules [36]. ROS concentrations is frequently quantified by measuring the amount of Malondialdehyde (MDA) and relative electrolytic leakage [37]. MDA content and relative electrolytic leakage of the leaves from wild-type plants and two *sapk3* mutant lines were compared. After drought treatment, two *sapk3* mutant lines accumulated more MDA than the wild-type plants (Fig. 5 C). Moreover, relative electrolytic leakage of two *sapk3* mutant lines was higher than that in wild-type plants (Fig. 5 D). Given the increased ROS-induced damage of *sapk3* mutants, the activities of catalase (CAT), superoxide dismutase (SOD), peroxidase (POD) and the content ascorbic acid (AsA) from wild-type plants and two *sapk3* mutant lines were also measured. Compared with wild-type plants, two *sapk3* mutant lines showed lower CAT, SOD, POD activities and AsA content than wild-type plants under drought conditions (Fig. 5 F-I).

**Fig. 6:**
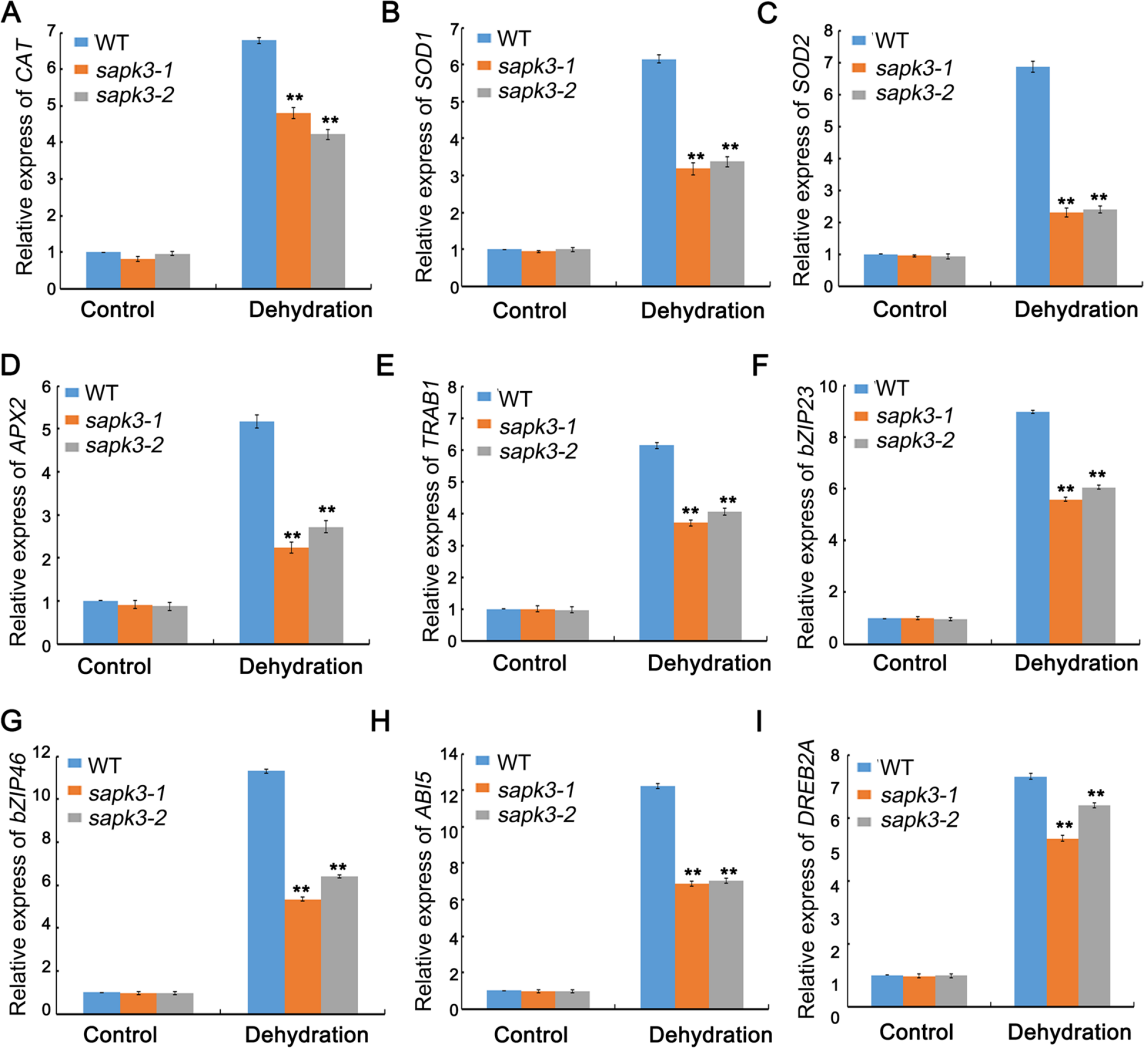
The expression of abiotic stress-responsive genes. **A**-**I** Relative expression levels of *OsCAT*, *OsSOD1*, *OsSOD2*, *OsAPX2*, *OsTRAB1*, *OsbZIP23, OsbZIP46*, *OsABI5* and *OsDREB2A* from the seedings of 2-week-old wild-type plants and two *sapk3* mutant lines before and after drought stress. Values represent the means ± SD of three biological replicates (5 plants for each replicate). **P < 0.01 (Student’s t-test)

To determine whether *OsSAPK3* is involved in ROS-scavenging, the expression levels of several antioxidant genes including *OsCAT*, *OsSOD1*, *OsSOD2* and *ascorbic acid peroxidase 2* (*OsAPX2*) were assessed. The expression levels of these genes were significantly lower in two *sapk3* mutant lines than that in wild-type plants (Fig. 6A-D). These results demonstrated that *OsSAPK3* was also important for improving ROS tolerance by regulating ROS detoxification.

### OsSAPK3 involved in drought stress response in ABA dependent and independent manner

In order to explore the possible mechanism of *OsSAPK3* in the regulation of drought stress, the expression of 5 well-characterized drought resistance-related genes (i.e., *OsTRAB1* [38], *OsABI5* [39], *OsbZIP23* [34], *OsbZIP46* [33] and *OsDREB2A* [40]) were analyzed. Among these genes, *OsTRAB1*, *OsABI5*, *OsbZIP23 and OsbZIP46* are involved in ABA dependent signaling, *OsDREB2A* is involved in ABA independent regulatory systems. All their expression levels were significantly lower in two *sapk3* mutant lines than that in wild-type plants after drought treatment (F ig. 6E-I). These results suggested that *OsSAPK3* upregulated the expression of some stress-responsive genes both in ABA-dependent and ABA-independent manner under drought stress.

### *OsSAPK3* improved rice yield by altering tiller numbers and grain size

**Fig. 7:**
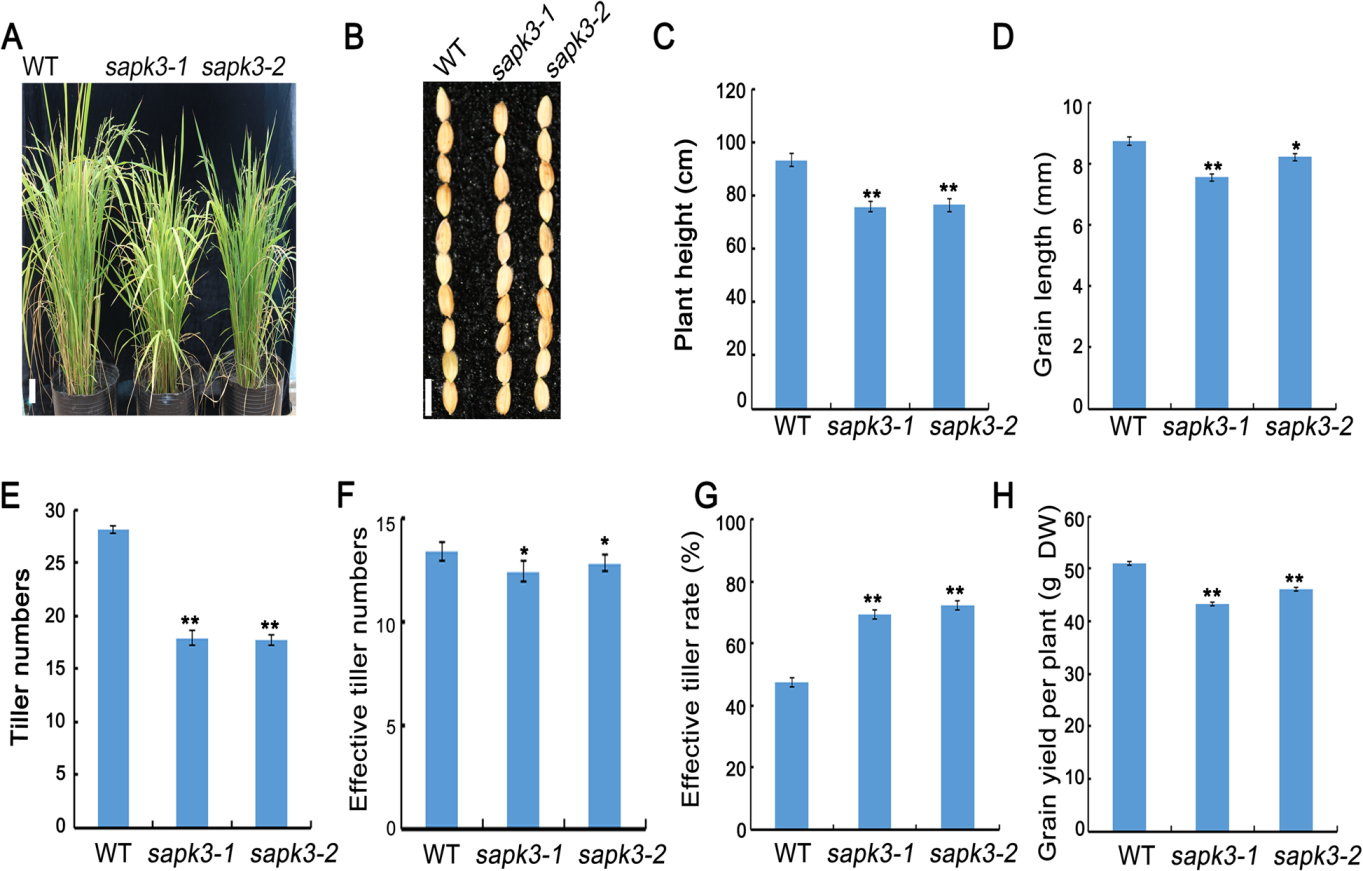
*OsSAPK3* affected growth phenotypes in rice **A** Growth phenotypes **B** Grain phenotypes **C** Plant height **D** Grain length **E** Tiller numbers **F** Effective tiller numbers **G** Effective tiller rate **H** Grain yield per plant of wild-type plants and two *sapk3* mutant lines.Values represent the means ± SD of three biological replicates (25 plants for each replicate). *P < 0.05 and **P < 0.01 (Student’s t-test)

In order to assess the effects of *OsSAPK3* on rice growth and productivity, the wild-type plants and two *sapk3* mutant lines were grown in the field. At maturity, compared to wild-type plants, plant height of two *sapk3* mutant lines decreased significantly (Fig. 7 A and C). In addition to dwarf phenotype, two *sapk3* mutant lines showed significantly fewer tiller numbers than wild-type plants (Fig. 7 E). However, effective tiller numbers of *sapk3* mutant plants were slightly fewer than wild-type plants (Fig. 7 F). The effective tiller rate of *sapk3* mutants was significantly higher than wild-type plants (Fig. 7 G). These results indicated that *OsSAPK3* participated in the regulation of rice tillering.

**Fig. 8:**
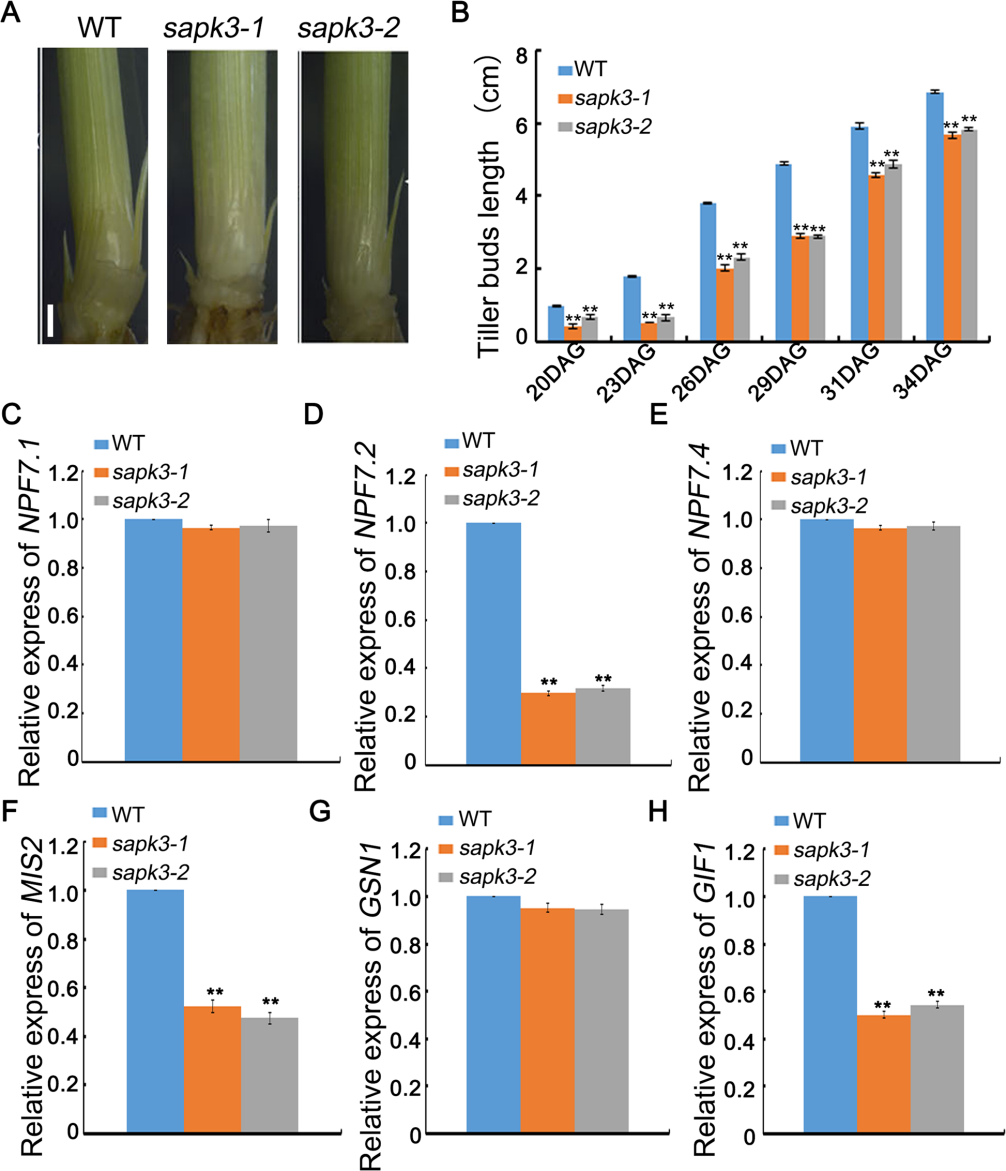
*OsSAPK3* altered tiller bud outgrowth in rice **A** Comparison of tiller buds at 20 days after germination among wild-type plants and two *sapk3* mutant lines. bar = 1 cm. **B** Statistical analysis at different days after germination among wild-type plants and two sapk3 mutant lines. Values represent the means ± SD of 3 biological replicates (25 plants for each replicate). **C** Expression levels of *OsNPF7.1* **D** *OsNPF7.2* **E** *OsNPF7.4* **F** *OsMIS2,* **G** *OsGSN1* and **H** *OsGIF1* among wild-type plants and two *sapk3* mutant lines. Values represent the means ± SD of three biological replicates (5 plants for each replicate). **P < 0.01 (Student’s t-test)

The tiller number is one key factor determining rice grain yield. Efficient utilization of nitrogen (N) is one important factor affecting rice tillering [41]. So far, many nitrate transporter genes, such as nitrate and di/tripeptide transporters (NPFs), have been found in rice [42]. OsNPF7.1, OsNPF7.2 and OsNPF7.4 was reported to positively regulate the tiller number [41, 42]. Additionally, *OsSAPK2*, which is homologous to *OsSAPK3,* could enhance grain production by regulating nitrogen utilization efficiency [43]. The function of *OsSAPK3* on rice tillering was investigated by analysing tiller buds development of wild-type plants and two *sapk3* mutant lines. Tiller buds grew slower in two *sapk3* mutant lines than that in wild-type plants, and this phenomenon was observed continuously for 34 days after germination (DAG) (Fig. 8 A and B). Based on these results, it could be concluded that *OsSAPK3* promoted rice tiller bud outgrowth between 20 and 34 DAG. To investigate how *OsSAPK3* altered the tiller buds, the expression levels of *OsNPF7.1*, *OsNPF7.2* and *OsNPF7.4* were measured. These results showed that the expression level of *OsNPF7.2* in two *sapk3* mutant lines was significantly lower than that in wild-type plants (Fig. 8 D). However, expression levels of *OsNPF7.1* and *OsNPF7.4* exhibited no significant difference between wild-type plants and two *sapk3* mutant lines (Fig. 8 C and E). These results suggested that *OsSAPK3* positively regulated rice tillering by promoting rice tiller bud outgrowth through regulating expression of nitrate transporter genes.

On the other hand, rice yield is also estimated via panicle length, grain number, seed setting rate per panicle, grain size and grain yield per plant [1]. Analyses of panicle presented that there was no significant difference in panicle length, grain number and seed setting rate per panicle between two *sapk3* mutant lines and wild-type plants (Fig. S1 A and C-E). This result suggested that OsSAPK3 might not participate in the regulation of pollen fertility. In addition, there was no significant difference in grain width and 1000-seed weight (Fig. S1 B and F). Further analyses of grain size and yield revealed that grain length and grain yield per plant of two *sapk3* mutant lines were significantly lower than that of wild-type (Fig. 7 B, D and H).

Several genes related to grain size in rice have been identified, such as *GRAIN SIZE MINI SEED 2* (*MIS2*) [44], *AND NUMBER1*(*GSN1*) [45] and *GRAIN INCOMPLETE FILLING 1* (*GIF1*) [46]. In order to investigate how *OsSAPK3* changed grain length, the expression levels of *OsMIS2, OsGSN1* and *OsGIF1* were measured. These results demonstrated that the expression levels of *OsMIS* and *OsGIF1* in two *sapk3* mutant lines were significantly lower than that in wild-type plants (Fig. 8 F and H). But the expression level of *OsGSN1* showed no significant difference between two *sapk3* mutant lines and wild-type plants (Fig. 8 G). These results suggested that *OsSAPK3* regulated grain length by promoting expression levels of seed size related genes.

## Discussion

### *OsSAPK3* confers drought tolerance in an ABA-dependent manner in rice

Plant-specific kinases, SnRK2s are considered to be major regulators in the plant responses to challenging environmental conditions [17]. *OsSAPK2*, member of subclass II SnRK2s, is involved in the osmotic stress response by regulating the expression of ABA dependent genes [47, 48]. These reports suggest that subclass II SnRK2s play important roles in osmotic stress signaling and promotes us to explore the potential role of *OsSAPK3*. However, direct experimental evidence of the role of OsSAPK3 in abiotic stress is largely lacking.

In this study, sequence alignment showed that *OsSAPK3* differed dramatically from subclass III SnRK2s in terms of ABA-activated motif (marked with yellow lines) (Fig. 1 A). But it was highly similar to *AtSnRK2.8* in *A. thaliana* and *OsSAPK2* in rice (Fig. 1 B). SnRK2.8 is strongly activated by salt and mannitol, slightly activated by ABA [31]. *OsSAPK2* is strongly induced by drought, NaCl and PEG, but not by ABA in rice [49]. It is found that *OsSAPK3* was significantly induced by drought, NaCl and PEG stress, and weakly induced by ABA (Fig. 2 A). In addition, *OsSAPK3* was highly expressed in roots and weakly in leaves (Fig. 2 B and C) which was consistent with the expression patterns of *AtSnRK2.8* [31], but different from *OsSAPK2* [49]. These findings suggested that *OsSAPK3* might acquire functions different from subclass III SnRK2s but similar to O*sSAPK2* and *AtSnRK2.8* during long-term evolution.

Mizoguchi et al. report that *AtSnRK2.8* play important roles in drought stress signaling in *A. thaliana* [32]. Here, the results demonstrated that *OsSAPK3* was localized in the nucleus and cytoplasm (Fig. 2 D), which was quite similar to *AtSnRK2.8* [32]. In addition, the hypersensitivity to exogenous ABA reduced in *sapk3* mutants lines (Fig. 3 D-F). Further research showed that *sapk3* mutants exhibited decreased drought tolerance (Fig. 4 A), as charactered by lower survival rate (Fig. 4 B) and higher water loss rate under drought conditions (Fig. 4 C). Under environmental stresses, ABA induces stomatal closure through the activation and inactivation of ion channels, such as SLAC1 and SLAC7 in *A. thaliana* [50, 51]. In rice, SAPK8 has been found to activate the function of OsSLAC1 [52]. SAPK2 [29] and SAPK9 [53] positively regulated the stomatal closure by up regulating the expression of *OsSLAC1* and *OsSLAC7*. Under drought conditions, stomatal conductance in *sapk3* mutant plants leaves was remarkably increased (Fig. 4E), and expression patterns of *SLAC1* and *SLAC7* in *sapk3* mutant plants leaves were remarkably decreased compared with wide type plants (Fig. 4 F and G). These results suggested that *OsSAPK3* functioned positively in drought stress response by ABA-dependent manner.

### *OsSAPK3* affects physiological and metabolic regulation in response to drought stress

In rice, understanding the physiological adaptation of drought resistance is very useful for developing high-yield varieties under drought conditions. Previous studies have shown that plant growth is severely restricted by abiotic stresses, mainly due to protein denaturation, lipid peroxidation, cell homeostasis destruction, cell oxidative damage and DNA mutations caused by the accumulation of ROS [54, 55]. Lipid peroxidation induced by ROS accumulation can damage cell membranes [55]. Therefore, relative ion leakage is a good indicator to evaluate membrane stability under cold, drought or salt stress conditions in rice and wheat [56, 57]. The results in this study revealed that MDA content and relative ion leakage in *sapk3* mutants were significantly higher than that in wild-type plants (Fig. 5 C and D), suggesting that *OsSAPK3* played a crucial role in alleviating oxidative damage in rice.

According to previous reports, ROS detoxification plays a key role in drought and heat stress response [58, 59]. The antioxidant system that protects plants against adverse effects of ROS contains enzymatic antioxidants and non enzymatic molecules. Enzymatic antioxidants include SOD, CAT and APX [60]. AsA serves as non enzymatic antioxidant [60]. Therefore, CAT, SOD, POD activities and AsA content can be used as important indicators for drought tolerance in plants. In this study, activities of CAT, SOD, POD and AsA content of two *sapk3* mutant lines were significantly lower than that in wild-type plants (Fig. 5 F-I). To understand the molecular mechanism of decreased ROS levels and higher antioxidant activities in two *sapk3* mutant lines, the expression levels of *OsCAT*, *OsSOD1*, *OsSOD2* and *OsAPX2* were tested*.* In this study, the expression levels of *OsCAT*, *OsSOD1*, *OsSOD2* and *OsAPX2* exhibited significant downregulation in two *sapk3* mutant lines (Fig. 6 A-D), which was consistent with lower CAT, SOD, POD activities and AsA content in two *sapk3* mutant lines (Fig. 5 F-I). These results supported that *OsSAPK3* regulated the accumulation of ROS through altering the expression of ROS-scavenging genes under drought stress. ABA not-activated SnRK2s, such as SnRK2.4 and SnRK2.10, take part in ROS production/accumulation under salt or high osmotic stress conditions in *A. thaliana* [61]. In rice, OsSAPK2 improved ROS detoxification by promoting the generation of ROS scavengers under salt or drought stress [33, 42]. SAPK9 decreased cellular oxidative damage by reducing ROS accumulation [53]. These results suggested that *OsSAPK3* could be involved in the SnRK2-dependent ROS production and scavenging.

Early studies showed that under dehydration conditions, the accumulation of compatible osmoregulation substances such as proline and soluble sugar could certify the normal metabolic pathway by providing osmotic balance [54]. Under drought stress, plants can repair damage by accumulating proline to increase antioxidant activity [34]. Therefore, proline content can be used as an important reference for drought resistance. In transgenic rice, overexpression of *OsP5CS* showed a great improvement for drought tolerance [55]. SAPK2 [29] and SAPK9 [53] positively regulates drought or salt stress tolerance by increasing the osmotic adjustment and stomatal closure in rice. To test if *OsSAPK3* plays a role in proline and soluble sugar accumulation under drought stress, proline and soluble sugar contents were examined. Under drought condition, contents of proline and soluble sugar in two *sapk3* mutant lines were significantly reduced compared with wild-type plants (Fig. 5 A and B). Along with this, relative expression of *OsP5CS* was significantly reduced in two *sapk3* mutant lines (Fig. 5 E). These results implied that *OsSAPK3* functioned positively by influencing accumulation of compatible osmolytes in drought stress response.

Subclass III SnRK2s phosphorylate and activate several transcription factors, such as ABA-responsive element binding factor (ABF/AREB) transcription factors *OsTRAB1* (*OsbZIP66*) [38], *OsABI5* [39], *OsbZIP23* [34] and *OsbZIP46* [33]. Dehydration responsive element-binding protein 2A (*OsDREB2A)* mediates transcriptional changes to acquire stress resistance in ABA-independent pathways [40]. In this study, the transcript levels of *OsTRAB1*, *OsABI5*, *OsbZIP23* and *OsbZIP46* were all significantly lower in two *sapk3* mutant lines than in wild-type plants under drought conditions (Fig. 6 E–H). In addition, the analyses of *OsDREB2A* expression level showed that there was also significantly decreased in two *sapk3* mutant lines than in wild-type plants under drought conditions (Fig. 6I). As ABA-responsive bZIP transcription factors, OsbZIP23 and OsbZIP46 can be activated by ABA-dependent SnRKs including SAPK2 [29], SAPK6 [29] and SAPK9 [53]. These results suggested that *OsSAPK3* had several substrates in both ABA-dependent and ABA-independent signaling.

In total, *OsSAPK3* might play an important role in both ABA-dependent and ABA-independent drought stress signaling pathways.

### *OsSAPK3* improved rice yield by altering tiller numbers and grain length

It has reported that overexpression of SRK2C/SnRK2.8 enhances plant growth in *A. thaliana*, which may due to the phosphorylation of enzymes involved in metabolic processes [31]. SAPK9 increases rice yield by influencing panicle weight and spikelet fertility [53]. Under drought stress in reproductive period, *sapk2* mutant showed dwarf phenotype, with decreased grain number per panicle and grain yield per plant [40]. To gain more comprehensively understanding of *OsSAPK3*, plants height between wild-type plants and two *sapk3* mutant lines was evaluated. The plant height of two *sapk3* mutant lines decreased significantly (Fig. 7 A and C). These results implied that *OsSAPK3* had a similar function to *AtSnRK2.8* or *OsSAPK2* in regulating plant growth.

Previous studies have reported that effective tillers per plant is an essential determinant of rice yield, which is affected by genetic and environmental factors [2]. *TE* controls tillering and shoot branching [6]. In addition, *TE-OE* lines have much fewer tillers, whereas the *loss-of-function te* mutants have much more tillers [6]. More interesting, subclass III SAPKs (SAPK8, SAPK9 and SAPK10) are involved in shoot branching and tillering by inhibiting APC/C^TE^ activity through phosphorylating TE [7]. In addition to dwarf phenotype, tiller numbers and effective tillers reduced significantly in two *sapk3* mutant lines (Fig. 7 E and F). These results indicated that, similar to subclass III SAPKs, *OsSAPK3* might participated in the regulation of rice tillering.

In previous research, *OsSAPK2* can improve grain yield by adjusting nitrogen utilization efficiency [40]. Rice contains a large gene family, which is used for the uptake and transport of nitrate and small peptides. For example, *OsNPF7.1*, *OsNPF7.1* or *OsNPF7.4* participate in the regulation of crop yield by promoting the growth of axillary buds and by increasing the number of tillers [38]. Overexpression of *OsNPF7.1*, *OsNPF7.1* and *OsNPF7.4* could enhance grain yield by improving the uptake of nitrate [39]. In order to further explore its possible regulatory mechanism, the development of tiller buds and the expression levels of *OsNPF7.1*, *OsNPF7.2* and *OsNPF7.4* between two *sapk3* mutant lines and wild-type plants were investigated. These results showed that tiller buds grew shorter in two *sapk3* mutant lines (Fig. 8 A and B). In addition, the expression level of *OsNPF7.2* in two *sapk3* mutant lines was significantly lower than that in wild-type plants (Fig. 8 D). These results suggested that *OsSAPK3* regulated rice tiller numbers by affecting the growth of axillary buds via promoting expression of nitrate transporter genes.

On the other hand, results from analysis of other agronomic traits showed that, compared with wild-type plants, grain length and grain yield per plant decreased significantly in two *sapk3* mutant lines (Fig. 7 D and H). However, there is no significant change in other traits, such as grain width, panicle length, grain number per panicle, seed setting rate per panicle and 1000-seed weight (Fig. S1 A-F). Grain size is directly associated with grain yield in rice. In rice, loss-of-function of *GSN1* caused larger grains by decreasing cell number due to reduced cell division during spikelet development [45]. *MIS2* controls grain size by regulating epidermal cell size and cell number. In *mis2* mutant, the grain showed reduced length, width and thickness [44]. Overexpression of *GIF1*, which encodes a cell-wall invertase, increased grain size [46]. In this study, the expression levels of *OsMIS* and *OsGIF1* in two *sapk3* mutant lines was significantly lower than that in wild-type plants (Fig. 8 F and H). These results suggested that *OsSAPK3* regulated grain length by promoting expression levels of seed size related genes. In conclusion, these results demonstrated that *OsSAPK3* improved rice yield by participating in the regulation of tiller numbers and grain size indirectly.

## Conclusions

In this study, *OsSAPK3* was significantly induced by drought, NaCl and PEG stress, and weakly induced by ABA. *OsSAPK3* was localized in the nucleus and cytoplasm. In order to characterize the role of *OsSAPK3* in drought stress response, *OsSAPK3* mutated plants (*sapk3-1* and *sapk3-2*) was generated using CRISPR/Cas9 system. *sapk3-1* and *sapk3-2* seedings reduced the hypersensitivity to exogenous ABA. In addition, *sapk3-1* and *sapk3-2* showed decreased stress tolerance, accompanied by lower survival rates, higher water loss rate, increased stomatal conductance and remarkably decreased expression of *SLAC1* and *SLAC7* under drought stress*.* These results suggested that *OsSAPK3* functioned positively in drought stress response by ABA-dependent manner. In addition, physiological and metabolic analyses showed that *OsSAPK3* played an important role in both ABA dependent and independent abiotic stress signaling pathway, including influencing accumulation of compatible osmolytes, ROS detoxification, expression of ABA dependent and independent dehydration-responsive genes. Results of agronomic traits demonstrated that *OsSAPK3* improved rice yield by regulating tiller numbers and grain size. The experimental of tiller bud development suggested that *OsSAPK3* regulated rice tiller numbers by affecting axillary bud growth via promoting the expression of nitrate transporter genes. Further studies suggested that *OsSAPK3* regulated grain length by promoting expression levels of seed size related genes. These findings can be used to improve rice yield under drought stress. However, the in-depth mechanism needs further study.

## Materials and methods

### Plant cultivation and agronomic traits analyses

Wild-type rice plants (*Oryza sativa L.*) used in this paper, were from the laboratory of XiaoYan Yang, Faculty of Life Science and Technology, Kunming University of Science and Technology.

All seeds of different genotypes were harvested at the same time and stored in the same environment. Seeds of wild-type plants and two *sapk3* mutant lines germinated on 1/2 Murashige and Skoog (MS) medium simultaneously for one week.

For ABA sensitivity assay, one week seedlings were transplanted into 1/2 MS medium containing 100 μM ABA. After 10 days treatment, shoot and root height were measured.

For abiotic stress experiment, one week seedlings were transplanted into the pot with same amount of soil, The drought phenotype of two weeks seedlings was identified after withdrawing water for 7 days and re-watering for 7 days.

For basic agronomic traits analysis, rice plants were grown in experimental plot of rice in Yuxi Normal University from March to August. Ten plants at a spacing of 16.5 cm × 26.5 cm were planted in a row and 5 rows of each line were planted. At reproductive stage, 25 plants of wild-type plants and two *sapk3* mutant lines were randomly chosen to detect agronomic traits.

### Generation and screening of transgenic plants

*OsSAPK3* mutants were generated by the CRISPR/Cas9 system. The CRISPR/Cas9 plasmid was designed according to the protocol described previously [62]. Concisely, the first and second exons of SAPK2 were selected for guide RNA design. Double-strand DNA generated by annealing the oligo pairs, and then was cloned into the pYLCRISPR/Cas9Pubi-H vector. Wild-type rice plants (*Oryza sativa L.*) was used for transformation. Then transgenetic seedlings were kept in growth chamber at 28^◦^C under long-day conditions (14 h light/10 h dark cycles). For mutation detection, genomic DNA extracted from mutant seedlings (all plant) were used for PCR. Then PCR products (sequence is in Supplementary Table 3) were identified by comparing the 19-bp gRNA target sequences (gtttcgagggggccaatgg, gattgatgaaaatgtgcag) to the rice reference genome (sequence is in Supplementary Table 3).

Based on mutation detection results, we selected two independent homozygous mutant lines in the T_1_ generation, which are named *sapk3-1* and *sapk3-2*. The primers used for CRISPR/Cas9 (U6a-*SAPK3*-F, U6a-*SAPK3*-R, U3-*SAPK3*-F, U3-*SAPK3*-R) and mutation detection (Cas9-*SAPK3*-F and Cas9-*SAPK3*-R) were listed in the Supplementary Table 3. Nucleotide sequence of SAPK3 CDS and predicted amino acid sequences in different genotypes were listed in the Supplementary Table 2.

### Phylogenetic tree and alignment

Sequences of selected SnRK2II and SnRK2III members in *A. thaliana* (At), *Oryza sativa* (Os) and *Zea mays* (Zm) were aligned with CLUSTALX (Sequences were listed in the Supplementary Table 1) [16, 63]. The phylogenic tree was constructed by MEGA 5.0. The reliability for the internal branch was evaluated by the bootstrap with 1000 bootstrap replicates and marked above the nodes.

### RNA extraction and qRT-PCR analyses

To detect the transcript level of target genes under different stresses, wild-type plants were kept in pot with same soil in growth chamber at 28^◦^C under long-day conditions (14 h light/10 h dark cycles) for 2 weeks. Two weeks old seedlings were treated with 100 μM ABA, 175 mM NaCl or 20% PEG 6000 (m/v) for 24 h. For drought stress, two weeks old seedlings were exposed to the air without water supply for 24 h. Leaves were sampled every 3 h.

For the qRT-PCR analysis, total RNA was isolated from rice seedlings using the TriZol reagent (Invitrogen). The cDNAs were obtained by using Superscript II in accordance with manufacturer’s instructions (Invitrogen). The qRT-PCR analysis was performed using SYBR Premix Ex Taq kit (Takara). The primers used in qRT-PCR analysis were listed in Supplementary Table 3.

### GUS Staining of *OsSAPK3*

For GUS reporter analysis, *OsSAPK3* promoter was amplified using primers Promoter-*SAPK3*-F and Promoter-*SAPK3*-R (Supplementary Table 3). The fused ProSAPK3-GUS was cloned into the P1300 vector. For the GUS staining analysis, leaves, stems and roots were destained with pure ethanol and then examined. The GUS activity was detected using the GUS blue kit (Huayueyang, Beijing, China) according to the manufacturer’s manual.

### Subcellular localization

For the transient expression assay in rice protoplasts, the full-length *OsSAPK3* CDS sequences were inserted into the 35S: GFP (PAN580) empty vector to generate the green fluorescent protein (GFP) recombinant vector 35S-*SAPK3*-GFP (pAN580). The 35S-GFP empty vector and the 35S-*SAPK3*-GFP recombinant vector were inserted into separate rice protoplasts as previously described [64]. Fluorescent signals were detected using the Zeiss LSM 710 laser scanning confocal microscope.

### Determination of metabolites

Water loss rate, relative ion leakage, the content of proline, soluble sugar and malondialdehyde (MDA) were checked following the method as previously described [49].

For water loss rate, flag leaves were detached and left on the laboratory bench at room temperature. Then weighed every 20 min. Water loss rate was expressed as a percentage of initial fresh weight.

For relative ion leakage, About 1 g flag leaves separated from different lines were cut into 5 mm length and placed in test tubes containing 10 ml deionized water. The tubes were covered with plastic caps and placed in a water bath maintained at the constant temperature of 22 °C for 2 h. The conductance of H_2_O was measured by conductivity meter (HORIBA TWIN COND B-173).

For the content of proline, about 0.5 g dried leaf segments from different lines were ground into powder with liquid nitrogen, and then homogenized with 10 ml of 3% sulphosalicylic acid in tube. Collected the supernatant after centrifuging for 20 min (3000 × g). 2 ml of supernatant was reacted with 2 ml acid ninhydrin and 2 ml glacialacetic acid in a test tube at 100^◦^C for 1 h. Then cooled on ice. The absorbance at 520 nm was measured by spectrophotometer.

Total soluble sugar content in leaves was determined using anthrone reagent. Approximately 0.5 g dried leaf segments from different lines were ground into powder with liquid nitrogen, and then homogenized with 2 ml 80% ethanol in shaker at 200 rpm for 1 h. Following centrifuge at 6,000 × g for 10 min, and then collected as much supernatant as possible. Added equal volume of chloroform, completely mix, and then centrifuged at 12,000 × g for 10 min. The aqueous part was transferred to a new tube, 50 μl of each was mixed with 4.95 ml anthrone reagent and then boiled for 15 min. Measured the optical density of glucose standards at 620 nm by spectrophotometer.

For the content of MDA, about 1 g leaf segments from different lines were homogenized in 10 ml of 10% trichloroacetic (v/v) and centrifuged at 5,000 × g for 10 min. Following 2 ml of supernatant was reacted with 2 ml thiobarbituric acid in a test tube at 100^◦^C for 15 min, quickly cooled on ice, and the absorbance at 532 nm was measured by spectrophotometer.

The activities of SOD, POD, CAT and AsA content were detected using copper-zinc superoxide dismutase (CuZn-SOD) assay kit (A001-4–1), peroxidase assay kit (A084-3–1) catalase (CAT) assay kit (A007-1–1) and Vitamin C assay kit (A009-1–1) (http://www.njjcbio.com/, Nanjing Jiancheng Bioengineering Institute) according to the manufacturer’s manual.

Two-week-old seedlings of two *sapk3* mutant lines and wide type plants withheld water for 7 days. According to the manual, leaf segments (0.5 g) from control and dehydration lines were homogenized using a chilled mortar.

For total SOD activity, 0.5 g leaf segments were ground into homogenate with 0.2 ml phosphate buffer solution (0.1 mol/L pH7-7.4), and then homogenized with 0.2 ml reagent No. 7. Centrifuged at 4,000 × g for 15 min. Then collected supernatant for Cu/Zn SOD determination. 2 ml of supernatant was reacted with 0.1 ml reagent No. 1, No. 2, No. 3 and No. 4 in a test tube at 37^◦^C for 40 min. Then reacted with 2 ml chromogenic agent for 10 min at room temperature. Measured the absorbance at 550 nm.

For POD activity, 0.5 g leaf segments were ground into homogenate with 0.2 ml phosphate buffer solution (0.1 mol/L pH7-7.4). Then collected the supernatant after centrifuging for 10 min (3500 × g). 0.1 ml of supernatant was reacted with 2.4 ml reagent No. 1, 0.3 ml reagent No. 2 and 0.2 ml reagent No. 3 in a test tube at 37^◦^C for 30 min. Then reacted with reagent No. 4 for 10 min at 37^◦^C. Collected the supernatant after centrifuging for 10 min (3500 × g). Measure the absorbance at 420 nm.

For CAT activity, 0.5 g leaf segments were ground into homogenate with 4.5 ml phosphate buffer solution (0.1 mol/L pH7-7.4). Then collected the supernatant after centrifuging for 10 min (2000 × g). 0.05 ml supernatant was reacted with 1 ml reagent No. 1 and 0.1 ml reagent No. 2 in a test tube at 37^◦^C for 1 min. Then mixed with 1 ml reagent No. 3 and 0.1 ml reagent No. 4. Measured the absorbance at 405 nm.

For the content of ascorbic acid (AsA), 0.5 g leaf segments were ground into homogenate with 5 mL of 5% (w/v) m-phosphoric acid. Following centrifugation at 10,000 × g for 15 min at 4 °C, the supernatant was used for the determination of AsA. Following 2 ml of supernatant was reacted with specified dosage of R1, R2, R3 and R4 (According to the manual) in a test tube at 22 ◦C for 30 min, quickly cooled on ice, and the absorbance at 536 nm was measured.

### Stomatal number and stomatal conductance analysis

Leaves from two weeks wild-type plants and two *sapk3* mutant lines were fixed with 2.5% glutaraldehyde and observed by scanning electron microscopy (S-3400 N, Hitachi, Japan). The second leaves of two weeks rice plants were selected for the stomatal conductance analysis, which was performed using the LI-6400XT Portable Photosynthesis System (LI-COR, USA) before and after 7 days exposure to drought stress.

### Statistical analyses

Three independent experiments were conducted for drought stress analysis and gene expression analyses. Three biological replicates were performed for each experiment. For agronomic traits analyses, 25 plants of wild-type plants or two *sapk3* mutant lines were randomly chosen to detect agronomic traits. Excel 2010 was used for making charts. All results are presented as means ± standard derivation (SD) of three biological replicates. Statistically significant difference analysis was conducted by “Student’s t-test” using SPSS statistics software.

## Supplementary Information

**Additional file 1: Supplementary Table 1. **Amino acid sequences of selected*SnRK2s* inthe phylogenetic tree.

**Additional file 2: Supplementary Table 2. **Nucleotide sequence of *SAPK3* CDS and predicted amino acid sequences in different genotypes.

**Additional file 3: Supplementary Table 3.** Primers and oligos used in this study.

**Additional file 4: Supplementary Figure 1. **Agronomic traits of *sapk3*mutant lines. (A) Panicle phenotypes of wild-type plants and two *sapk3* mutant lines. (B) Grain width (C) Panicle length (D) Grain number per panicle (E) Setting rate per panicle (F) 1000-seed weight of wild-type plants and two *sapk3* mutant lines. Values represent the means ± SD of three biological replicates (25 plants for each replicate).

## Data Availability

All data generated or analyzed during this study are included in this published article and its supplementary information files. The datasets and materials used or analysed during the current study available from the corresponding author on reasonable request.
