# Supplementary material for: Molecular characterization reveals that OsSAPK3 improves drought tolerance and grain yield in rice
Source: BMC Plant Biol. 2023 Jan 24;23:53. doi: 10.1186/s12870-023-04071-8 (PMC9872327; doi:10.1186/s12870-023-04071-8)
Supplement: Supplementary file 1 — Additional file 1: Supplementary Table 1. Amino acid sequences of selectedSnRK2s inthe phylogenetic tree. [file 12870_2023_4071_MOESM1_ESM.doc]

**Supplementary Table 1 Amino acid sequences of selected *SnRK2s* in the phylogenetic tree.**

| **Species name** | **Gene name** | **Amino acid sequence** |
| --- | --- | --- |
| Arabidopsis thaliana (At) | ATsnrk2.2 AT3G50500 | M D P A T N S P I M P I D L P I M H D S D R Y D F V K D I G S G N F G V A R L M T D R V T K E L V A V K Y I E R G E K I D E N V Q R E I I N H R S L R H P N I V R F K E V I L T P S H L A I V M E Y A A G G E L Y E R I C N A G R F S E D E A R F F F Q Q L I S G V S Y C H A M Q I C H R D L K L E N T L L D G S P A P R L K I C D F G Y S K V L F I S L K S S V L H S Q P K S T V G T P A Y I A P E I L L R Q E Y D G K L A D V W S C G V T L Y V M L V G A Y P F E D P Q E P R D Y R K T I Q R I L S V T Y S I P E D L H L S P E C R H L I S R I F V A D P A T R I T I P E I T S D K W F L K N L P G D L M D E N R M G S Q F Q E P E Q P M Q S L D T I M Q I I S E A T I P T V R N R C L D D F M A D N L D L D D D M D D F D S E S E I D V D S S G E I V Y A L |
|  | ATsnrk2.3 AT5G66880 | M D R A P V T T G P L D M P I M H D S D R Y D F V K D I G S G N F G V A R L M R D K L T K E L V A V K Y I E R G D K I D E N V Q R E I I N H R S L R H P N I V R F K E V I L T P T H L A I I M E Y A S G G E L Y E R I C N A G R F S E D E A R F F F Q Q L L S G V S Y C H S M Q I C H R D L K L E N T L L D G S P A P R L K I C D F G Y S K S S V L H S Q P K S T V G T P A Y I A P E V L L R Q E Y D G K I A D V W S C G V T L Y V M L V G A Y P F E D P E E P R D Y R K T I Q R I L S V K Y S I P D D I R I S P E C C H L I S R I F V A D P A T R I S I P E I K T H S W F L K N L P A D L M N E S N T G S Q F Q E P E Q P M Q S L D T I M Q I I S E A T I P A V R N R C L D D F M T D N L D L D D D M D D F D S E S E I D I D S S G E I V Y A L |
|  | ATsnrk2.6 AT4G33950 | M D R P A V S G P M D L P I M H D S D R Y E L V K D I G S G N F G V A R L M R D K Q S N E L V A V K Y I E R G E K I D E N V K R E I I N H R S L R H P N I V R F K E V I L T P T H L A I V M E Y A S G G E L F E R I C N A G R F S E D E A R F F F Q Q L I S G V S Y C H A M Q V C H R D L K L E N T L L D G S P A P R L K I C D F G Y S K S S V L H S Q P K S T V G T P A Y I A P E V L L K K E Y D G K V A D V W S C G V T L Y V M L V G A Y P F E D P E E P K N F R K T I H R I L N V Q Y A I P D Y V H I S P E C R H L I S R I F V A D P A K R I S I P E I R N H E W F L K N L P A D L M N D N T M T T Q F D E S D Q P G Q S I E E I M Q I I A E A T V P P A G T Q N L N H Y L T G S L D I D D D M E E D L E S D L D D L D I D S S G E I V Y A M |
|  | ATsnrk2.7 AT4G40010 | M E R Y D I L R D L G S G N F G V A K L V R E K A N G E F Y A V K Y I E R G L K I D E H V Q R E I I N H R D L K H P N I I R F K E V F V T P T H L A I V M E Y A A G G E L F E R I C N A G R F S E D E G R Y Y F K Q L I S G V S Y C H A M Q I C H R D L K L E N T L L D G S P S S H L K I C D F G Y S K S S V L H S Q P K S T V G T P A Y V A P E V L S R K E Y N G K I A D V W S C G V T L Y V M L V G A Y P F E D P E D P R N I R N T I Q R I L S V H Y T I P D Y V R I S S E C K H L L S R I F V A D P D K R I T V P E I E K H P W F L K G P L V V P P E E E K C D N G V E E E E E E E E K C R Q S V E E I V K I I E E A R K G V N G T D N N G G L G L I D G S I D L D D I D D A D I Y D D V D D D E E R N G D F V C A L |
|  | ATsnrk2.8 AT1G78290 | M E R Y E I V K D I G S G N F G V A K L V R D K F S K E L F A V K F I E R G Q K I D E H V Q R E I M N H R S L I H P N I I R F K E V L L T A T H L A L V M E Y A A G G E L F G R I C S A G R F S E D E A R F F F Q Q L I S G V N Y C H S L Q I C H R D L K L E N T L L D G S E A P R V K I C D F G Y S K S G V L H S Q P K T T V G T P A Y I A P E V L S T K E Y D G K I A D V W S C G V T L Y V M L V G A Y P F E D P S D P K D F R K T I G R I L K A Q Y A I P D Y V R V S D E C R H L L S R I F V A N P E K R I T I E E I K N H S W F L K N L P V E M Y E G S L M M N G P S T Q T V E E I V W I I E E A R K P I T V A T G L A G A G G S G G S S N G A I G S S S M D L D D L D T D F D D I D T A D L L S P L |
| Oryza sativa (Os) | OsSAPK1 LOC_Os03g27280 | M E R Y E V M R D I G S G N F G V A K L V R D V A T N H L F A V K F I E R G L K I D E H V Q R E I M N H R S L K H P N I I R F K E V V L T P T H L A I V M E Y A A G G E L F E R I C N A G R F S E D E A R F F F Q Q L I S G V S Y C H S M Q V C H R D L K L E N T L L D G S V T P R L K I C D F G Y S K S S V L H S Q P K S T V G T P A Y I A P E V L S R K E Y D G K V A D V W S C G V T L Y V M L V G A Y P F E D P D D P R N F R K T I T R I L S V Q Y S I P D Y V R V S A D C R H L L S R I F V G N P E Q R I T I P E I K N H P W F L K N L P I E M T D E Y Q R S M Q L A D M N T P S Q S L E E V M A I I Q E A R K P G D A M K L A G A G Q V A C L G S M D L D D I D D I D D I D I E N S G D F V C A L |
|  | OsSAPK2 LOC_Os07g42940 | M E R Y E V I K D I G S G N F G V A K L V R D V R T K E L F A V K F I E R G Q K I D E N V Q R E I M N H R S L R H P N I V R F K E V V L T P T H L A I V M E Y A A G G E L F E R I C S A G R F S E D E A R F F F Q Q L I S G V S Y C H S M Q I C H R D L K L E N T L L D G S I A P R L K I C D F G Y S K S S L L H S Q P K S T V G T P A Y I A P E V L A R K E Y D G K V A D V W S C G V T L Y V M L V G A Y P F E D P D E P R N F R K T I T R I L S V Q Y M V P D Y V R V S M E C R H L L S R I F V A N P E Q R I T I P E I K N H P W F L K N L P I E M T D E Y Q M S V Q M N D I N T P S Q G L E E I M A I I Q E A R K P G D G S K F S G Q I P G L G S M E L D D V D T D D I D V E D S G D F V C A L |
|  | OsSAPK3 LOC_Os10g41490 | M E E R Y E A L K E L G A G N F G V A R L V R D K R S K E L V A V K Y I E R G K K I D E N V Q R E I I N H R S L R H P N I I R F K E V C L T P T H L A I V M E Y A A G G E L F E Q I C T A G R F S E D E A R Y F F Q Q L I S G V S Y C H S L E I C H R D L K L E N T L L D G S P T P R V K I C D F G Y S K S A L L H S K P K S T V G T P A Y I A P E V L S R E E Y D G K V A D V W S C G V T L Y V M L V G S Y P F E D P G D P R N F R K T I S R I L G V Q Y S I P D Y V R V S S D C R R L L S Q I F V A D P S K R I T I P E I K K H T W F L K N L P K E I S E R E K A D Y K D T D A A P P T Q A V E E I M R I I Q E A K V P G D M A A A D P A L L A E L A E L K S D D E E E A A D E Y D T Y |
|  | OsSAPK8 LOC_Os03g55600 | M A A A G A G A G A P D R A A L T V G P G M D M P I M H D S D R Y E L V R D I G S G N F G V A R L M R D R R T M E L V A V K Y I E R G E K I D D N V Q R E I I N H R S L K H P N I I R F K E V I L T P T H L A I V M E Y A S G G E L F E R I C K N V R F S E D E A R Y F F Q Q L I S G V S Y C H S M Q V C H R D L K L E N T L L D G S P A P R L K I C D F G Y S K S S V L H S Q P K S T V G T P A Y I A P E V L L K K E Y D G K T A D V W S C G V T L Y V M V V G A Y P F E D P E E P K N F R K T I Q R I L N V Q Y S I P E N V D I S P E C R H L I S R I F V G D P S L R I T I P E I R S H G W F L K N L P A D L M D D D S M S S Q Y E E P D Q P M Q T M D Q I M Q I L T E A T I P P A C S R I N H I L T D G L D L D D D M D D L D S D S D I D V D S S G E I V Y A M |
|  | OsSAPK9 LOC_Os12g39630 | M E R A A A G P L G M E M P I M H D G D R Y E L V K E I G S G N F G V A R L M R N R A S G D L V A V K Y I D R G E K I D E N V Q R E I I N H R S L R H P N I I R F K E V I L T P T H L A I V M E Y A S G G E L F E R I C S A G R F S E D E A R F F F Q Q L I S G V S Y C H S M Q V C H R D L K L E N T L L D G S T A P R L K I C D F G Y S K S S V L H S Q P K S T V G T P A Y I A P E V L L K K E Y D G K I A D V W S C G V T L Y V M L V G A Y P F E D P E D P K N F R K T I Q K I L G V Q Y S I P D Y V H I S P E C R D L I T R I F V G N P A S R I T M P E I K N H P W F M K N I P A D L M D D G M V S N Q Y E E P D Q P M Q N M N E I M Q I L A E A T I P A A G T S G I N Q F L T D S L D L D D D M E D M D S D L D L D I E S S G E I V Y A M |
|  | OsSAPK10 LOC_Os03g41460 | MDRAALTVGPGMDMPIMHDGDRYELVRDIGSGNFGVARLMRSRADGQLVAVKYIERGDKIDENVQREIINHRSLRHPNIIRFKEVILTPTHLAIVMEYASGGELFERICNAGRFSEDEARFFFQQLISGVSYCHSMQVCHRDLKLENTLLDGSTAPRLKICDFGYSKSSVLHSQPKSTVGTPAYIAPEVLLKKEYDGKIADVWSCGVTLYVMLVGAYPFEDPDEPKNFRKTIQRILGVQYSIPDYVHISPECRDLIARIFVANPATRISIPEIRNHPWFLKNLPADLMDDSKMSSQYEEPEQPMQSMDEIMQILAEATIPAAGSGGINQFLNDGLDLDDDMEDLDSDPDLDVESSGEIVYAM |
| Zea mays (Zm) | ZmSnRK2.1 AC199054.2 | MEYAAGGELFERICSAGRFSEDEARFFFQQLISGVSYCHSMQVCHRDLKLENTLLDGSVAPRLKICDFGYSKSSVLHSQPKSTVGTPAYIAPEVLSRKEYDGKVADVWSCGVTLYVMLVGAYPFEDPDDPKNFRKTITRILSVQYSIPDYVRVTMECGHLLSRIFVGNPEQRITIPEIKKHPWFLKNLPIEMTDEYQQSMQLADMNTPGQSLEEVMAIIQEARKPGDAMNLAGQLPCLGSVDLDDIDFDDIDDIDTENSGDFVCAV |
|  | ZmSnRK2.2 AC186803.4 | MERYEVIKDIGSGNFGVAKLVRDVRTKELFAVKFIERGMKIDENVQREIMNHRSLRHPNIVKFKEVVLTPTHLAIVMEYAAGGELFERICNAGRFSEDEARFFFQQLISGVSYCHSMQICHRDLKLENTLLDDSIAPRLKICDFGYSKSSVLHSQPKSTVGTPAYIAPEVLARKEYDGKVADVWSCGVTLYVMLVGAYPFEDPDEPKNFRKTLTRIISVQYAVPDFVRVSMECRHLLSRIFVAKPEQRITIPEIKNHPWFLKNLPIEMTDEYQMNLQLVDMNVPSQSLEEIMSIILEARKPGDGLKHAGQLPGLGSMELDDIDVDDIDVEDSGDFVCAL |
|  | ZmSnRK2.3 AC196411.3 | MEERYEALKELGAGNFGVARLVRDKRTKELVAVKYIERGKKIDENVQREIINHQSLRHPNIVRFKEVCLTPTHLAIVMEYAAGGELFEKICSAGRFSEDESRYFFQQLISGVSYCHSMEICHRDLKLENTLLDGSPTPRVKICDFGYSKSALLHSKPKSTVGTPAYIAPEVLSRKEYDGKVADVWSCGVTLYVMLVGSYPFEDPEDPRNFRKTISRILGVQYSIPDYVRVSSDCRRLLSQIFVADPSKRITIPEIKHHPWFLKNLPREISEREKANYKDADAAEPAQAVDEIMRIVEEAKTPGDMSKVVDPALLAEMAELESDEEEADADDTY |
