# Supplementary material for: Molecular characterization reveals that OsSAPK3 improves drought tolerance and grain yield in rice
Source: BMC Plant Biol. 2023 Jan 24;23:53. doi: 10.1186/s12870-023-04071-8 (PMC9872327; doi:10.1186/s12870-023-04071-8)
Supplement: Supplementary file 2 — Additional file 2: Supplementary Table 2. Nucleotide sequence of SAPK3 CDS and predicted amino acid sequences in different genotypes. [file 12870_2023_4071_MOESM2_ESM.doc]

**Supplementary Table 2 Nucleotide sequence of *SAPK3* CDS and predicted amino acid sequences in different genotypes**

| **Genotype** | **Nucleotide sequence of *SAPK3* CDS** |
| --- | --- |
| **WT** | atggaggagaggtacgaggcgttgaaggagctcggggccggcaacttcggggtggccaggctggtcagggacaagaggagcaaggagctcgtcgccgtcaagtacatcgagaggggcaagaagattgatgaaaatgtgcagagggagatcatcaatcataggtcgctccggcatcccaatatcattcggtttaaggaggtttgtttgacacccacacacctagccattgtcatggagtatgctgctggtggagaactctttgaacaaatctgcaccgcagggcgattcagcgaagacgaggcaaggtacttcttccagcagctaatatcaggtgtcagctactgtcattctctggaaatttgccaccgtgatcttaaacttgagaacaccctcctggatggaagcccaacacctcgtgtgaagatttgtgactttggttactcaaagtctgctttgctgcattcgaagccgaagtctacagttggtactccagcatacatagcgccagaagttctttcaagagaagaatatgatggcaaggtagcagacgtttggtcctgtggtgtgacactgtacgtgatgcttgtcggttcatacccgtttgaagatccaggtgatccgaggaatttccgcaaaacgatcagcagaattcttggcgtgcaatactccatcccggactacgtgagggtgtcttccgactgcaggcgccttctatctcaaatatttgttgccgatccttcaaagaggatcacgatccctgagataaagaagcacacgtggtttctgaagaatctgccaaaggagatatcggagagggagaaggccgactacaaggacacggacgccgcccctccgacgcaggccgtcgaggagatcatgcggatcatccaggaggccaaggtccccggcgacatggccgccgccgacccggcgctgctcgcggagctcgccgagctgaagagcgacgacgaagaggaggccgccgatgagtatgacacctactga |
| ***sapk3-1*** | Atgggtacgaggcgttgaaggagctcggggccggcaacttcggggtggccaggctggtcagggacaagaggagcaaggagctcgtcgccgtcaagtacatcgagaggggcaagaagattgatgaaaatgtgcagagggagatcatcaatcataggtcgctccggcatcccaatatcattcggtttaaggaggtttgtttgacacccacacacctagccattgtcatggagtatgctgctggtggagaactctttgaacaaatctgcaccgcagggcgattcagcgaagacgaggcaaggtacttcttccagcagctaatatcaggtgtcagctactgtcattctctggaaatttgccaccgtgatcttaaacttgagaacaccctcctggatggaagcccaacacctcgtgtgaagatttgtgactttggttactcaaagtctgctttgctgcattcgaagccgaagtctacagttggtactccagcatacatagcgccagaagttctttcaagagaagaatatgatggcaaggtagcagacgtttggtcctgtggtgtgacactgtacgtgatgcttgtcggttcatacccgtttgaagatccaggtgatccgaggaatttccgcaaaacgatcagcagaattcttggcgtgcaatactccatcccggactacgtgagggtgtcttccgactgcaggcgccttctatctcaaatatttgttgccgatccttcaaagaggatcacgatccctgagataaagaagcacacgtggtttctgaagaatctgccaaaggagatatcggagagggagaaggccgactacaaggacacggacgccgcccctccgacgcaggccgtcgaggagatcatgcggatcatccaggaggccaaggtccccggcgacatggccgccgccgacccggcgctgctcgcggagctcgccgagctgaagagcgacgacgaagaggaggccgccgatgagtatgacacctactga |
| ***sapk3-2*** | atggaggagaggtacgaggcgttgaaggagctcggggccggcaacttcggggtggccaggctggtcagggacaagaggagcaaggagctcgtcgccgtcaagtacatcgagaggggcaagaagattgagggagatcatcaatcataggtcgctccggcatcccaatatcattcggtttaaggaggtttgtttgacacccacacacctagccattgtcatggagtatgctgctggtggagaactctttgaacaaatctgcaccgcagggcgattcagcgaagacgaggcaaggtacttcttccagcagctaatatcaggtgtcagctactgtcattctctggaaatttgccaccgtgatcttaaacttgagaacaccctcctggatggaagcccaacacctcgtgtgaagatttgtgactttggttactcaaagtctgctttgctgcattcgaagccgaagtctacagttggtactccagcatacatagcgccagaagttctttcaagagaagaatatgatggcaaggtagcagacgtttggtcctgtggtgtgacactgtacgtgatgcttgtcggttcatacccgtttgaagatccaggtgatccgaggaatttccgcaaaacgatcagcagaattcttggcgtgcaatactccatcccggactacgtgagggtgtcttccgactgcaggcgccttctatctcaaatatttgttgccgatccttcaaagaggatcacgatccctgagataaagaagcacacgtggtttctgaagaatctgccaaaggagatatcggagagggagaaggccgactacaaggacacggacgccgcccctccgacgcaggccgtcgaggagatcatgcggatcatccaggaggccaaggtccccggcgacatggccgccgccgacccggcgctgctcgcggagctcgccgagctgaagagcgacgacgaagaggaggccgccgatgagtatgacacctactga |
|  |  |
| **Genotype** | **Amino acid sequence of SAPK3** |
| **WT** | M E E R Y E A L K E L G A G N F G V A R L V R D K R S K E L V A V K Y I E R G K K I D E N V Q R E I I N H R S L R H P N I I R F K E V C L T P T H L A I V M E Y A A G G E L F E Q I C T A G R F S E D E A R Y F F Q Q L I S G V S Y C H S L E I C H R D L K L E N T L L D G S P T P R V K I C D F G Y S K S A L L H S K P K S T V G T P A Y I A P E V L S R E E Y D G K V A D V W S C G V T L Y V M L V G S Y P F E D P G D P R N F R K T I S R I L G V Q Y S I P D Y V R V S S D C R R L L S Q I F V A D P S K R I T I P E I K K H T W F L K N L P K E I S E R E K A D Y K D T D A A P P T Q A V E E I M R I I Q E A K V P G D M A A A D P A L L A E L A E L K S D D E E E A A D E Y D T Y * |
| ***sapk3-1*** | M G T R R * |
| ***sapk3-2*** | M E E R Y E A L K E L G A G N F G V A R L V R D K R S K E L V A V K Y I E R G K K I E G D H Q S * |
