# Supplementary material for: Molecular characterization reveals that OsSAPK3 improves drought tolerance and grain yield in rice
Source: BMC Plant Biol. 2023 Jan 24;23:53. doi: 10.1186/s12870-023-04071-8 (PMC9872327; doi:10.1186/s12870-023-04071-8)
Supplement: Supplementary file 3 — Additional file 3: Supplementary Table 3. Primers and oligos used in this study. [file 12870_2023_4071_MOESM3_ESM.doc]

**Supplementary Table 3 Primers and oligos used in this study**

| Name | Primer sequence (5’-3’) |
| --- | --- |
| **Plasmid Constrcutions and Mutation Detection** | |
| U6a-*SAPK3*-F | GCCGgtttcgagggggccaatgg |
| U6a-*SAPK3*-R | caaagctcccccggttaccCAAA |
| U3-*SAPK3*-F | GGCAgattgatgaaaatgtgcag |
| U3-*SAPK3*-R | ctaactacttttacacgtcCAAA |
| Cas9-*SAPK3*-F | GCTTCAAGTCCGACCAAGAGAC |
| Cas9-*SAPK3*-R | AATCAGCCAGGGTAAGCTTTGT |
| Promoter-*SAPK3*-F | GCAAGACGCAACAAGAAGATGC |
| Promoter-*SAPK3*-R | ATAGGATCCTGGCCCCCTCGAAA |
| P30-*SAPK3*-F | ATGGAGGAGAGGTACGAGGCGTTGAA |
| P30-*SAPK3*-R | TCAGTAGGTGTCATACTCATCGGCGGC |
| ***qRT-PCR*** | |
| *OsActin*-RT-F | AGCTGCGGGTATCCATGAGA |
| *OsActin*-RT-R | GCAATGCCAGGGAACATAGTG |
| *OsP5CS1*-RT-F | GCTGACATGGATATGGCAAAAC |
| *OsP5CS1*-RT-R | GTAAGGTCTCCATTGCATTGCA |
| *OsTRAB1*-RT-F | TTTCCCACATTCATATTGATGTTAA |
| *OsTRAB1*-RT-R | TCCACATTCATATTGATGTTAATGA |
| *OsbZIP23*-RT-F | GGAGCTGAACGATGAACTCCAG |
| *OsbZIP23-*RT-R | TCGGCTCATTCTCTCTAGAACCTC |
| *OsbZIP46*-RT-F | AACAAGAAAGCGTCCCCACA |
| *OsbZIP46-*RT-R | CGAGCTCCACCGTATAAGCC |
| *OsABI5*-RT-F | GGGAATGGGATGATGATTGAGA |
| *OsABI5*-RT-R | CCACATGGGAGAGGACAATACC |
| *OsDREB2A*-RT-F | CTGATAGCCTCCTTGATTTT |
| *OsDREB2A*-RT-R | AAGACGAAAACCGTAAATG |
| *OsSLAC1*-RT-F | ATCACCAAGGACAGGCAGAACG |
| *OsSLAC1*-RT-R | TGATGTCGTACACCCTCTTGCC |
| *OsSLAC7*-RT-F | TACGCTTGCAAGGTGGTCTT |
| *OsSLAC7*-RT-F | GGCGCGAAGAAGAAGTTGAC |
| *OsCAT*-RT-F | TACTTCCCATCCCGCTACGA |
| *OsCAT*-RT-R | TCCTTACATGCTCGGCTTCG |
| *OsSOD1*-F | CAGGTTGAGGGAGTCGTCAC |
| *OsSOD1*-R | GGTTGCCTCAG CTACACCTT |
| *OsSOD2*-F | GTGAAGGCTGTTGTTGTGCT |
| *OsSOD2*-R | GCCAGAGACACTTCCAGTCA |
| *OsAPX2*-RT-F | TCCTACGCCGACTTCTACCA |
| *OsAPX2*-RT-R | CGGCGTAATCCGCAAAGAAG |
| *OsNPF7.1*-RT-F | AAGGGCGGGTGGATCACGTTCCCT |
| *Os NPF7.1*-RT-R | ACGCTGATGCAGCCGGAGACGAT |
| *OsNPF7.2*-RT-F | TGCAAGTGCCACTCCTCAAGG |
| *Os NPF7.2*-RT-R | AGGACGGTCTCCAGGTACACCACC |
| *OsNPF7.4*-RT-F | ACTCCAGGACGACGTCAGCCT |
| *Os NPF7.4*-RT-R | ACCAGGTTCGTGGCAATGCCGT |
| *OsMIS2*-RT-F | AGAGGTCATCTAACCATTCGTG |
| *OsMIS2*-RT-R | CCTCTGAGATGAAGTCACACTT |
| *OsGSN1*-RT-F | AGTATCTCAGAGGCTTGCGGTAA |
| *OsGSN1*-RT-R | AGCACTTCCAAAAGATTCTCCGT |
| *OsGIF1*-RT-F | ATCACCAACGGGAAGATATCTC |
| *OsGIF1-*RT-R | GTTATTGAAAACGTAAAGGCGC |
