# Supplementary material for: Molecular characterization reveals that OsSAPK3 improves drought tolerance and grain yield in rice
Source: BMC Plant Biol. 2023 Jan 24;23:53. doi: 10.1186/s12870-023-04071-8 (PMC9872327; doi:10.1186/s12870-023-04071-8)
Supplement: Supplementary file 4 — Additional file 4: Supplementary Figure 1. Agronomic traits of sapk3mutant lines. (A) Panicle phenotypes of wild-type plants and two sapk3 mutant lines. (B) Grain width (C) Panicle length (D) Grain number per panicle (E) Setting rate per panicle (F) 1000-seed weight of wild-type plants and two sapk3 mutant lines. Values represent the means ± SD of three biological replicates (25 plants for each replicate). [file 12870_2023_4071_MOESM4_ESM.docx]

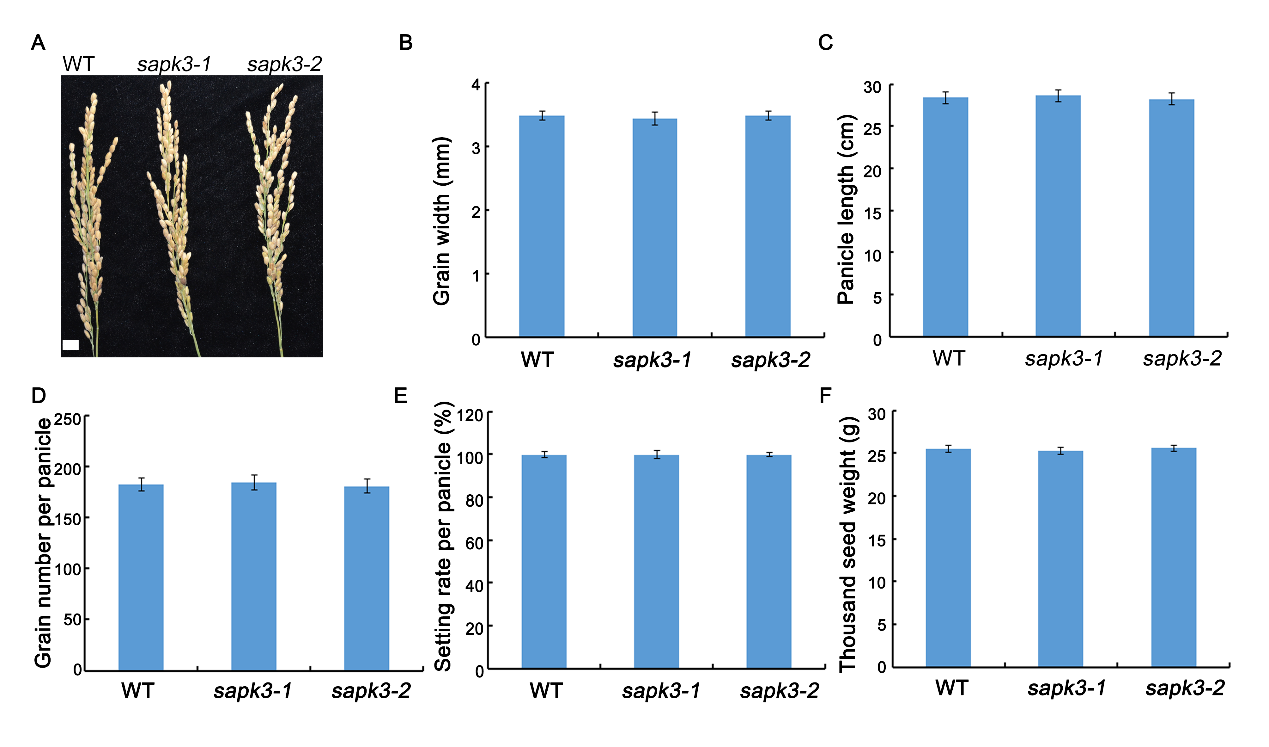


**Supplementary figure 1 Agronomic traits of *sapk3* mutant lines**

(A) Panicle phenotypes of wild-type plants and two *sapk3* mutant lines. (B) Grain width (C) Panicle length (D) Grain number per panicle (E) Setting rate per panicle (F) 1000-seed weight of wild-type plants and two *sapk3* mutant lines. Values represent the means ± SD of three biological replicates (25 plants for each replicate).
